# A Novel Equipment for VLE Studies: Ethanol and Water System at Moderate Pressures and Data Verification with a Direct Point-to-Point Test

**DOI:** 10.1021/acsomega.6c04827

**Published:** 2026-08-05

**Authors:** Pedro Susial Badajoz, Isabel Garcia Montesdeoca, Dunia E. Santiago

**Affiliations:** Universidad de Las Palmas de GranCanaria, 16750 Instituto de Estudios Ambientales y Recursos Naturales (iUNAT), Las Palmas de Gran Canaria, Canary Islands 35017, Spain

## Abstract

A new multipurpose rectification tower was tested. Made of stainless steel, this two-section piece of equipment is filled with Raschig rings. It can be used to study vapor–liquid equilibrium (VLE) and determine and verify azeotropes in mixtures, as well as the vapor pressures of pure substances. The tower was designed to enable both external recirculation of the phases and internal countercurrent circulation, generating intimate contact between nonmiscible phases. Due to the material it is made of, it can be operated at pressures other than atmospheric pressure. The degradation of the fluids’ quality can also be prevented. Experimental data obtained at 101, 300, 500, and 700 kPa using ethanol and water mixtures were verified by consulting bibliographic sources and applying the point-to-point test of Fredenslund et al. The reorganized errors observed when applying the point-to-point test suggested developing a new verification routine using the differential form of the Gibbs–Duhem equation. This new VLE validation procedure was tested using bibliographic data and was found to be more restrictive than the point-to-point test.

## Graphical abstract

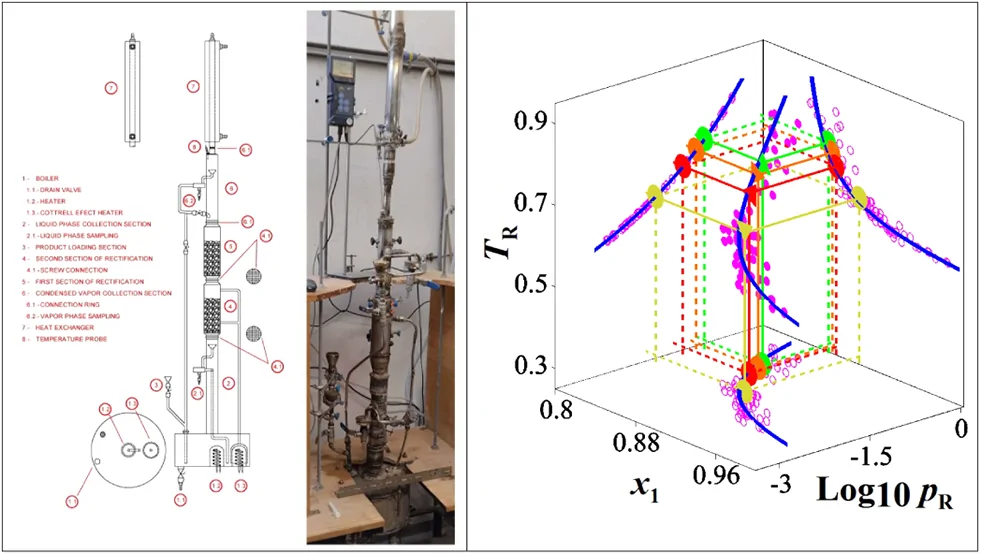

## 1 Introduction

In general, dynamic equipment in which circulation occurs in both phases is preferred for experimental determinations of the isobaric vapor–liquid equilibrium (VLE) of miscible substances. The fundamental challenge, however, lies in achieving significant speed in the circulation of both phases, as well as ensuring adequate interaction and contact.

The methods and equipment used in VLE experimental determinations are described and referenced in the bibliography.^1–6^ To avoid one of the main causes of errors in experimental determinations, the substances used must be of the highest purity. Another cause lies in the different types of equipment used. Most ebulliometers are made of glass. Consequently, they have typically been used for pressures below approximately 400 kPa and at temperatures ranging from 300 to 400 K. We are only aware of a few metal ebulliometers that have been used for moderate or high pressures.^7–11^

In previous papers, we described the design, construction, and testing of new metal ebulliometers made of copper^12^ and stainless steel.^13^ These ebulliometers and their various modifications have been used to obtain VLE data at different pressures, including high pressures.^14–16^ They were built to work, considering the Cottrell pump effect. Consequently, the mass transfer process takes place in cocurrent flow, meaning that the equilibrium condition can only be obtained after infinite contact time.

Therefore, bearing this in mind, we have built a new stainless steel equipment that is classified within the group of devices that work by recirculating both phases. The multipurpose equipment operates in parallel and countercurrent flow simultaneously. Furthermore, it is a rectification column designed to sample both phases simultaneously. Both characteristics distinguish it from the various devices described in the literature.^1–14^

Using oxygenated compounds, such as ethanol, has been suggested as a way of improving the octane rating of petrol. However, the substance must be purified to obtain it under anhydrous conditions. Several combined techniques have been suggested for achieving this. It is also possible to do so by operating under different pressure conditions (pressure–swing distillation). Therefore, it is important to evaluate the behavior and evolution of the VLE of ethanol and water mixtures at different pressures.

Conversely, a large amount of VLE data is available for the ethanol + water system under both isothermal and isobaric conditions.^17^ However, only a limited set of data is available for moderate and higher pressures.^7,17,18^ We recently obtained VLE data for the ethanol + water mixture at high pressures^19^ of 1500 and 2000 kPa through experimentation. In this study, VLE data at moderate pressures of 101, 300, 500, and 700 kPa were obtained to understand the evolution of the mixture and to evaluate the new multipurpose equipment.

In addition, the azeotrope for this system is described in the bibliography,^20^ although we also verified it.^19^ In this paper the singular point was also obtained to evaluate the new device. On the other side, the vapor pressures of pure water and ethanol can be found in the bibliography^17,19,21–23^ in both experimental and correlated data. Therefore, the vapor pressures of these substances were obtained to test the quality of the newly built multipurpose equipment.

The data obtained using the new equipment were verified using the usual consistency test,^24^ additionally, a new verification procedure was developed based on the Gibbs–Duhem differential equation and a symmetric activity coefficient model. The quality of the data can be observed without minimization of the recalculated properties. This new VLE verification procedure can be applied to binary mixtures of non–electrolyte homogeneous substances under low and moderate pressure and temperature conditions for isothermal and isobaric systems.

Consequently, the multipurpose equipment presented in this study was validated by determining the VLE data experimentally at different pressures and by conducting a conventional consistency test^24^ on the resulting data. Additionally, bibliographic^7,17–19^ data was used to verify the experimental results. The validation process also involved comparing how the single point of the mixture changes with pressure to the literature data.^19,20^ Finally, we used the vapor pressures obtained with the experimental equipment to validate the distillation tower in relation to the literature data.^7,19,21–23^

## 2 Experimental Section

### 2.1 Chemicals and Apparatus

Densities of pure components and their mixtures were measured with a Mettler Toledo DM-40 model digital densimeter with an accuracy of 0.0001 g/cm^3^.This equipment was calibrated with air and water. The refractive index of pure components was measured with an Atago RX-7000α digital refractometer with an accuracy of 0.0001. Water was employed to calibrate the refractometer.

Table 1 shows the physical properties, density (ρ_*ii*_), and refractive index (*n*_D_) at 298.15 K, as well as the normal boiling point (*T*_bp_) determined for water and ethanol. The water used, which had a conductivity of 18.2 MΩ·cm, was obtained using a Milli-Q EQ 7000 ultrapure water purification system. A comparison with values found in the bibliography^25–30^ is also included in Table 1.

**Table 1:** Experimental and Bibliographic Physical Properties of Pure Substances at 101 kPa^a^

Chemical name	CAS No.	Source	Purity (mass %)	Purification method	Analysis method	*T*_bp_ (K)	ρ (298.15 K) (kg m^–3^)	*n*_D_ (298.15 K)
Water	7732-18-5	–	–	Reverse Osmosis	–	373.15^c^	997.1^d^	1.33249^e^
		–	–	Distillation	–	373.15^f^	997.0^f^	1.33350^f^
		–	–	Distillation	–	373.15^g^	997.0^g^	1.3332^g^
		–	–	Deionised	–	373.15^h^	997.4^h^	1.33249^h^
Ethanol	64-17-5	Panreac Química S.A	99.9	None	GC^b^	351.45^c^	785.2^d^	1.35933^e^
		Merck Farma	99.8	None	GC^b^	352.07^f^	785.02^f^	1.35922^f^
		–	–	Distillation	–	351.45^i^	351.45^i^	1.3594^i^
		Merck Farma	99.5	None	–	351.56^j^	351.56^j^	1.3592^j^
		Acros	99.5	–	GC^b^	351.46^k^	351.46^k^	1.35939^k^

a Standard uncertainties u are u­(*p*) = 0.5 kPa, u­(*T*) = 0.01 K, u­(ρ) = 0.1 kg m^–3^, u­(*n*_D_) = 0.0001.

b Gas chromatograph informed by the suppliers.

c The combined expanded uncertainties U­(k = 2) are U­(*T*_bp_) = 0.06 K, U $\left(p_{i}^{0}\right)$ = 2 kPa.

d The combined expanded uncertainties U­(k = 2) are U­(*T*) = 0.02 K, U­(*p*) = 1 kpa, U­(ρ) = 0.2 kg m^–3^.

e The combined expanded uncertainties U­(k = 2) are U­(*T*) = 0.02 K, U­(*p*)=1 kPa, U­(*n*_D_)=0.0002.

f Ref 25.

g Ref 26.

h Ref 27.

i Ref 28.

j Ref 29.

k Ref 30.

For the binary system of ethanol (1) and water (2), the calibration curve of ethanol mole fraction (*x*_1_) versus density (*ρ*_*ij*_) was obtained as a previous step to VLE determinations. Samples of ethanol and water mixtures, ranging from 0 to 1 mole fraction, were prepared by mass. The airtight, stoppered glass bottles were used. An Entris-224i-1S balance from Sartorius Lab Instruments GmbH & Co. with an uncertainty of 10^–4^ g was used to determine the mass of the substances. The densities of the samples (*ρ*_*ij*_) were measured at 298.15 K.

### 2.2 Equipment and Procedure

The new multipurpose experimental equipment (see Figure 1) was employed to obtain the vapor pressures as well as the VLE data for the ethanol (1) + water (2) binary mixtures at 101, 300, 500, and 700 kPa. This new equipment, which is described in the following paragraphs, was included in an experimental installation.^16^ The other elements of the experimental installation, as well as the measurement and control devices, have been described previously.^16^ Namely, for temperature measurements, a Pt100 from Termocal was used alongside a digital display P655 from Dotsmann Electronic GmbH. For the calibration of the probe, three different temperatures were established using a thermostatic bath in which a calibrated standard (Pt100 SDL model 5385/100) was connected to an AC resistance (ASL model D250), with a temperature resolution of 1 mK. The estimated uncertainty for temperature measurement was 10 mK (from 0 to 473 K) and 100 mK (above 473 K). For real-time pressure measurement, a GENSPEC GS4200 transducer from ESI Tech. Ltd. with an operating range from 0 to 2.5 MPa and ±0.001 MPa uncertainty was used.

**Figure 1:**
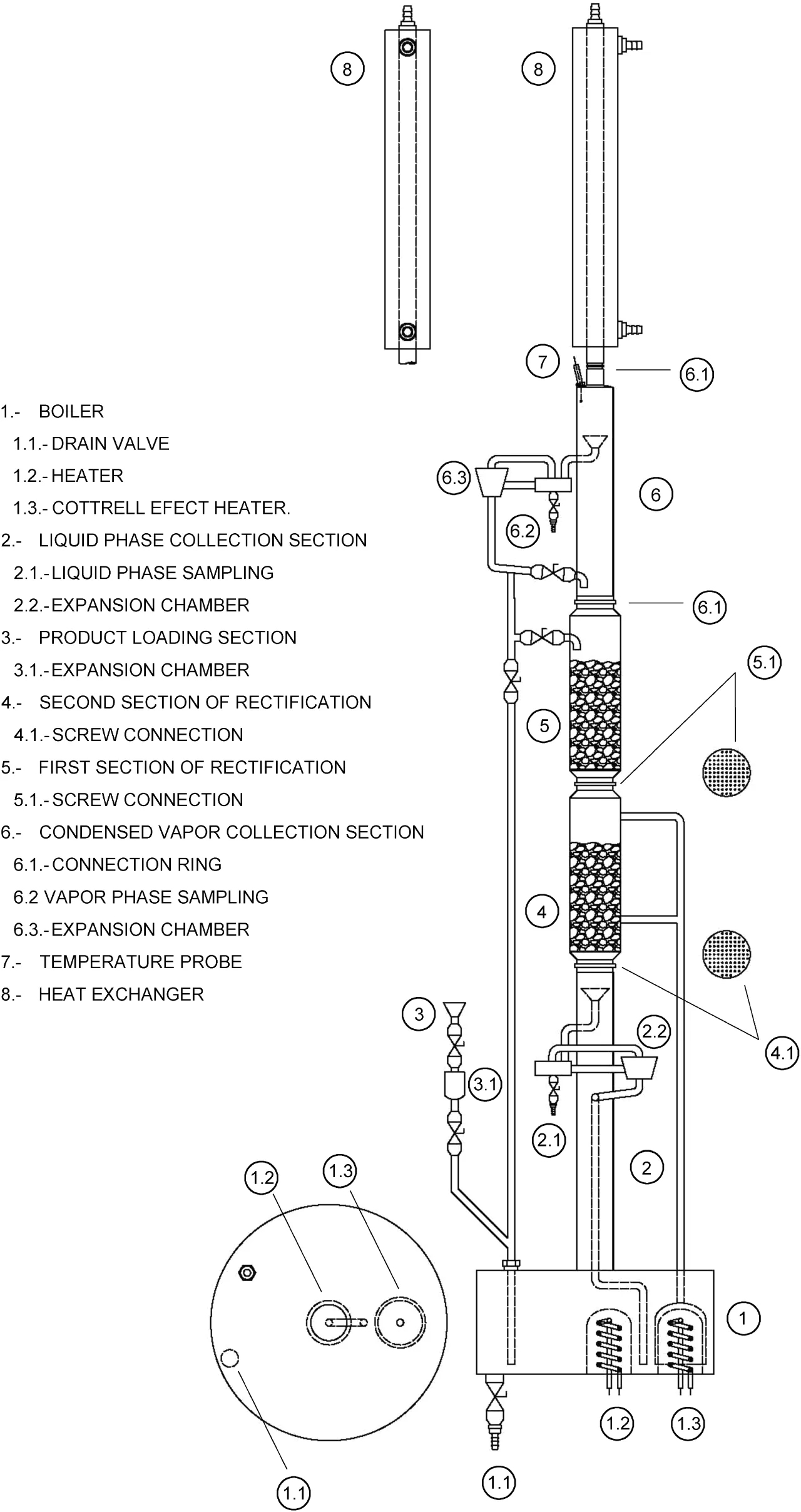
Distillation multipurpose equipment for VLE determinations.

The stainless steel equipment was made using a boiler {1} with an operating capacity of around 5000 cm^3^. The design of the boiler enables the dynamic recirculation of both phases due to the boiling effect by using an inverted vessel {1.2}. In addition, a double-walled inverted vessel was included to produce the Cottrell pump effect {1.3}. Therefore, the mass transfer process can be developed because of the intimate contact between the continuously recirculated phases from both the inverted vessel and the double-walled inverted vessel. By using these boiling chambers, it is possible to have both a cocurrent flow and a countercurrent flow of the substances.

To achieve cocurrent flow contact, a pipe was connected to an inverted double-walled vessel. To operate the phases in countercurrent flow, a distillation tower equipped with two rectification sections containing Raschig rings was connected to the boiler, as shown schematically in Figure 1.

The distillation tower has a liquid phase collection section {2}. This section contains a funnel cone with connections between the second rectification section {4} and the boiler {1}. The funnel is connected to a cylindrical vessel, and a pipe leading to the circulation of fluids inside the tower comes out of this vessel. Another pipe connected to the cylindrical vessel has a valve to take samples of the liquid phase {2.1}.

The second rectification section {4}, with a diameter of 65 mm, is located above the section for liquid phase collection. This section contains approximately 500 Raschig rings, each measuring 6 mm in diameter, which are arranged randomly. There is a lateral pipe for circulating the boiling mixture generated by the Cottrell pump.

The first rectification section {5} is welded to the second rectification section and has the same diameter and number of 6 mm Raschig rings. The condensed vapor collection section {6} is located at the top of the first rectification section.

The vapor collection section {6} has a funnel to collect the condensate, which is transferred to an external cylindrical vessel. This vessel contains a pipe with a ball valve to collect samples of the condensed vapor phase {6.2}, as well as a lateral pipe that ends in a T-connection with two bifurcations. One of the bifurcations of this lateral pipe is connected to a valve that returns the condensed vapor to the tower, while the other is connected by a valve to the product loading section.

The heat exchanger {8} to condense the vapor phase is located above the vapor-collection section. A welded connection is made between the vapor collection section and the heat exchanger.

The operating procedure is similar to that used for any distillation tower. First, the fluid is charged, and then the heating units are set on. After a while, the vapors generated move up the tower to the heat exchanger {8}. The condensed steam then circulates toward the tower and is channeled through a funnel, which is connected to a vessel where the condensed vapor phase is stored. The condensed vapor phase from this vessel can be sampled and can also be carried within through the Raschig rings in the first section of the tower. The vapor phase composition was determined by the densimetric technique described in Section 2.1.

Therefore, the descending liquid and the ascending vapor will come into countercurrent contact. This will facilitate the process of mass transfer between the phases. The heating in the double-walled inverted vessel circuit also makes the Cottrell pump effect possible. This generates an increase in the rising vapors in the tower along with the descending liquid in countercurrent through the second rectification section.

Therefore, the descending liquid and ascending vapor will be in countercurrent contact. Additionally, heating the mixture in the inverted double-walled vessel enables the Cottrell pump effect. This effect facilitates contact between phases in cocurrent flow contact. The boiling mixture introduced into the second rectification section by the Cottrell effect produces an ascending vapor that combines with the vapors from the boiler. Meanwhile, the descending boiling liquid from the Cottrell combines with the descending liquid from the first rectification section. This increases the amount of ascending vapor in the column and the amount of descending liquid in the second rectification section. These circumstances facilitate and increase the mass transfer process between the phases. This results from increased flow rates and better, more intimate contact between the circulating phases.

In the second rectification section, the circulating liquid comes into contact with the rising vapor. Subsequently, the descending liquid is collected by a funnel. The funnel is connected to a storage container. This container has two connections: one allows the liquid to be dispensed through a valve and the other circulates it to the boiler. The liquid phase composition was analyzed by using the calibration curve density vs composition described in Section 2.1.

In addition to the vapor–liquid equilibrium (VLE) process, manipulating the ball valves located outside the tower in the condensed vapor collection section {6} enables the total reflux operation. This allows the mixture to progress over time toward an equal composition in the liquid and vapor phases.

Conceptually, VLE data are obtained from a mass transfer process that develops between phases brought into contact. This process is dependent on the mass transfer coefficients and therefore on the contact velocity between the phases. In other words, it is dependent on the contact time between the phases. Therefore, the contact time needed to reach the steady state must be known (that is, when constant *T*, *p*, *x*, and *y* are observed). In practice, however, it is sufficient to know the residence time, from which statistically constant properties can be derived.

This process is a consequence of generating a disturbance in the composition of the contacting phases. Strictly speaking, there is no equality of chemical potentials between the phases. Once the necessary residence time has elapsed, the distribution of substances between the phases establishes a steady state, whereby the chemical potentials are equal.

To evaluate the residence time, a mixture of ethanol and water was prepared in a quantity greater than what was strictly required to operate the equipment, containing approximately 50% ethanol. The equipment was charged with this mixture and operated for 4 h to thermally homogenize the device and allow it to reach a constant temperature at atmospheric pressure. Then, samples of the phases were taken and analyzed. After this, the equipment was recharged with 30 cm^3^ of the initial mixture and operated for 30 min without changing the operating conditions. The phases were sampled and analyzed again. This procedure was repeated several times for the 30 min residence time.

The same procedure was repeated for different residence times. Analyzing the different phases, each with the same initial mixture composition and operating conditions but different residence times, revealed that steady-state conditions could be achieved with recirculation times of 2 h. Therefore, due to the disturbances generated in the mixture when unloading and loading substances into the equipment, it is necessary to leave the mixture operating inside the equipment for 2 h before proceeding with a new phase sampling. Similar techniques for determining vapor pressures and liquid–vapor equilibrium are described in the literature.^13,19^

## 3 Results and Discussion

### 3.1 Vapor Pressures

The experimental behavior of the equipment was first evaluated by determining the water and ethanol vapor pressures, considering the procedure mentioned above.^13,19^ Thus, the experimental *T* and $p_{i}^{0}$ values obtained (Table 2) for the pure substances were correlated to the Antoine equation,

1 $$\log_{10}\left(p_{i}^{0}/kPa\right)=A-B/\left(T/K-C\right)$$

and the standard deviations (*SD*) showed the quality of data regression. The deviation errors were calculated by comparison with the data obtained by using the Antoine constants of the literature^17,21–23^ presented in Table 3. The results are included in Figure 2. It can be observed that there is a good agreement between the experimental determinations of vapor pressures and calculated data.

**Table 2:** Experimental Vapor Pressures of Water and Ethanol^a^

$p_{i}^{0}\mathrm{/kPa}$	*T*/K	$p_{i}^{0}\mathrm{/kPa}$	*T*/K	$p_{i}^{0}\mathrm{/kPa}$	*T*/K	$p_{i}^{0}\mathrm{/kPa}$	*T*/K	$p_{i}^{0}\mathrm{/kPa}$	*T*/K
Water									
102	373.2	148	384.1	255	401.1	590	431.2	832	445.2
111	375.7	155	385.5	282	404.5	630	434.0	887	448.0
122	378.2	181	390.3	311	407.9	681	437.2	934	450.1
135	381.4	212	395.2	453	421.4	751	441.1	996	452.7
144	383.1	254	401.0	514	425.9	790	443.0		
Ethanol									
107	352.8	215	372.0	399	391.2	605	405.5	890	419.9
117	355.2	240	375.2	415	392.4	688	410.2	934	421.6
144	360.5	264	378.1	499	398.7	730	412.3	971	423.3
154	362.5	293	381.3	539	401.4	799	415.6	990	424.0
168	365.0	315	383.6	570	403.3	835	417.3	1015	425.1
193	368.9	344	386.4						

a The combined expanded uncertainties U­(k = 2) of temperature (*T*) and vapor pressures $\left(p_{i}^{0}\right)$ are U­(*T*) = 0.4 K, U $\left(p_{i}^{0}\right)$ = 0.003 MPa.

**Table 3:** Properties and the Antoine Constants Employed in the VLE of Ethanol (1) + Water (2) Treatment

Substance	*A* ^a^	*B* ^a^	*C* ^a^	SD $\left(p_{i}^{0}\right)$ kPa^a^	*SD*(*T*) K^a^	*A* ^b^	*B* ^b^	*C* ^b^	*A* ^c^	*B* ^c^	*C* ^c^	*A* ^d^	*B* ^d^	*C* ^d^	*A* ^e^	*B* ^e^	*C* ^e^
Water	7.20605	1765.486	33.377	2.15	0.14	7.19621	1730.630	39.724	7.06458	1650.910	46.793	7.05802	1648.806	46.815	7.09150	1673.310	44.163
Ethanol	6.99161	1460.701	58.477	1.11	0.07	7.32907	1642.890	42.85	6.89778	1390.869	67.060	6.91345	1393.500	67.757	7.01451	1475.739	56.818

a This work.

b Ref 17.

c Ref 21.

d Ref 22.

e Ref 23; $SD\left(F\right)=\sqrt{\frac{\overset{N}{\underset{1}{\sum }}{\left(F_{\exp}-F_{cal}\right)}^{2}}{N-2}}$ with *F* being $(p_{i}^{0}\mathrm{/kPa})$ or (*T*/K); *N* is the number of data.

**Figure 2:**
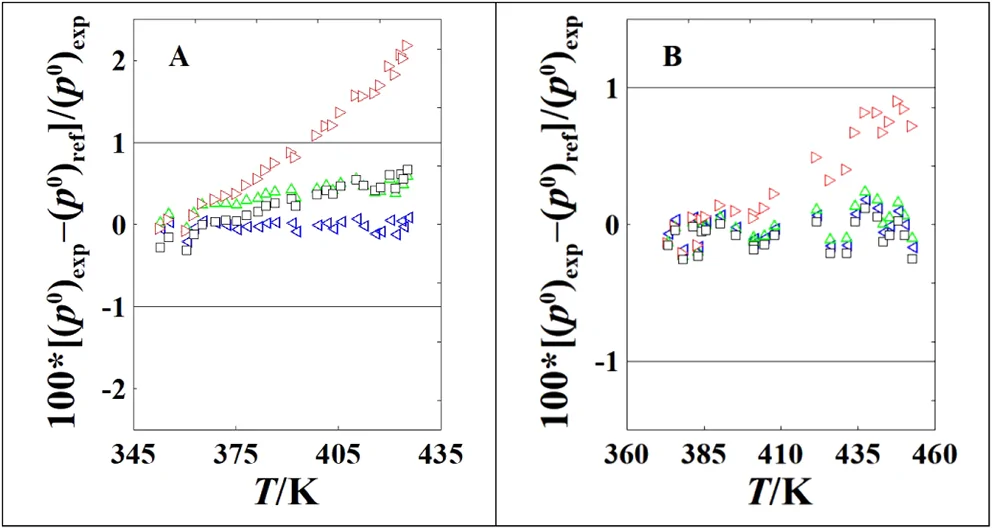
Plot of errors between experimental vapor pressures and bibliographic^17,21–23^ data: (A) ethanol (from 100 kPa to 1020 kPa range); (B) water (from 100 kPa to 1000 kPa range). Bibliographic data used are in ref 17 (red open right−pointing triangle), ref 21 (green open up−pointing triangle), ref 22 (black open square), ref 23 (blue open left−pointing triangle).

### 3.2 VLE Data Treatment

Previously, to determine the VLE data for the ethanol/water mixture, the densities of the binary alcohol/water mixtures studied in this work were measured at 298.15 ± 0.01 K. The ρ vs *x*_1_ data were correlated using the following polynomial equation:

2 $$\rho =\rho_{1}x_{1}+\rho_{2}\left(1-x_{1}\right)+x_{1}\left(1-x_{1}\right)\overset{m}{\underset{k=0}{\sum }}A_{k}{\left(\frac{x_{1}}{x_{1}+R_{V}\left(1-x_{1}\right)}\right)}^{k}$$

The correlation parameters are shown in Table 4. The experimental data for the binary mixtures of ethanol (1) and water (2) were verified alongside the values obtained for the excess volumes, and the results were checked against literature data.^19,31^ The deviation results showed no significant differences.

**Table 4:** Correlation of *ρ* vs *x*_1_ Pairs at 298.15 K and VLE Data with Fitting Coefficients and Standard Deviations

*FF*	*p*/kPa	*R*_*V*_	*A*_0_	*A*_1_	*A*_2_	*A*_3_	
Ethanol (1) + water (2) at 101 kPa							
Eq 2	101	0.31	–172.5	240.4	–518.7	363.7	*SD*(ρ/kg/m^3^) = 0.2
Eq 3	101	0.28	8.474	–20.825	19.289	–7.029	*SD*(*y*_1_–*x*_1_) = 0.002
Eq 4	101	0.34	–229.628	499.577	–413.93	120.329	*SD*(*T*/K) = 0.08
Eq 5	101	0.71	–18.796	126.142	–353.236	227.502	*SD*(*T*/K) = 0.19
Ethanol (1) + water (2) at 300 kPa							
Eq 3	300	0.19	9.151	–21.556	19.898	–7.609	*SD*(*y*_1_–*x*_1_) = 0.001
Eq 4	300	0.13	–305.908	445.951	–165.091		*SD*(*T*/K) = 0.03
Eq 5	300	1.76	3.752	–108.496	87.382		*SD*(*T*/K) = 0.14
Ethanol (1) + water (2) at 500 kPa							
Eq 3	500	0.24	6.889	–16.572	15.703	–6.147	*SD*(*y*_1_–*x*_1_) = 0.002
Eq 4	500	0.44	–220.659	488.444	–435.405	132.148	*SD*(*T*/K) = 0.12
Eq 5	500	0.41	–34.455	227.850	–522.601	295.765	*SD*(*T*/K) = 0.14
Ethanol (1) + water (2) at 700 kPa							
Eq 3	700	0.24	6.889	–16.572	15.703	–6.147	*SD*(*y*_1_–*x*_1_) = 0.002
Eq 4	700	0.44	–220.659	488.444	–435.405	132.148	*SD*(*T*/K) = 0.12
Eq 5	700	0.41	–34.455	227.850	–522.601	295.765	*SD*(*T*/K) = 0.14

After obtaining the calibration curve, the verification process of the new equipment was done by obtaining the VLE data corresponding to the ethanol/water binary mixtures at 101, 300, 500, and 700 kPa. These data are presented in Table 5. Data reduction was verified analytically by considering statistical deviations in the preceding stage prior to performing the thermodynamic treatment. The following polynomial equations were used to correlate the experimental data:

3 $$\left(y_{1}-x_{1}\right){[x_{1}\left(1-x_{1}\right)]}^{-1}=\overset{m}{\underset{k=0}{\sum }}A_{k}{\left(\frac{x_{1}}{x_{1}+R_{V}\left(1-x_{1}\right)}\right)}^{k}$$

4 $$\left[T-x_{1}T_{bp1}-\left(1-x_{1}\right)T_{bp2}\right]{[x_{1}\left(1-x_{1}\right)]}^{-1}=\overset{m}{\underset{k=0}{\sum }}A_{k}{\left(\frac{x_{1}}{x_{1}+R_{V}\left(1-x_{1}\right)}\right)}^{k}$$

5 $$\left[T-y_{1}T_{bp1}-\left(1-y_{1}\right)T_{bp2}\right]{[y_{1}\left(1-y_{1}\right)]}^{-1}=\overset{m}{\underset{k=0}{\sum }}A_{k}{\left(\frac{y_{1}}{y_{1}+R_{V}\left(1-y_{1}\right)}\right)}^{k}$$

**Table 5:** VLE Data at 101 kPa of Binary Mixtures^a^

*T/K*	*x*_1_	*y*_1_	γ_1_	γ_2_	*g* ^*E*^ */RT*	*T/K*	*x*_1_	*y*_1_	γ_1_	γ_2_	*g* ^*E*^ */RT*	*T/K*	*x*_1_	*y*_1_	γ_1_	γ_2_	*g* ^*E*^ */RT*
Ethanol (1) + water (2) at 101 kPa																	
370.60	0.011	0.097	4.390	0.989	0.005	353.75	0.381	0.611	1.460	1.292	0.303	351.65	0.782	0.805	1.015	2.009	0.163
368.35	0.022	0.178	4.348	0.988	0.020	353.45	0.412	0.618	1.382	1.352	0.311	351.50	0.824	0.839	1.009	2.069	0.136
365.65	0.037	0.259	4.131	0.998	0.051	353.20	0.444	0.631	1.321	1.395	0.309	351.45	0.849	0.856	1.001	2.163	0.117
363.00	0.059	0.347	3.811	0.994	0.074	353.05	0.469	0.641	1.278	1.430	0.305	351.41	0.866	0.871	1.000	2.187	0.105
362.25	0.065	0.366	3.748	1.000	0.086	352.95	0.498	0.655	1.234	1.460	0.295	351.37	0.874	0.878	1.001	2.204	0.100
361.75	0.071	0.384	3.665	0.996	0.089	352.70	0.516	0.664	1.219	1.490	0.295	351.35	0.884	0.886	0.999	2.239	0.093
360.05	0.090	0.432	3.459	1.001	0.113	352.70	0.544	0.671	1.169	1.549	0.284	351.31	0.894	0.895	0.999	2.261	0.086
359.05	0.105	0.462	3.288	1.002	0.127	352.60	0.572	0.684	1.137	1.592	0.272	351.30	0.899	0.899	0.999	2.284	0.082
358.35	0.113	0.475	3.223	1.014	0.145	352.40	0.605	0.701	1.110	1.646	0.260	351.32	0.910	0.909	0.997	2.308	0.072
357.20	0.134	0.501	2.992	1.032	0.175	352.20	0.629	0.715	1.097	1.684	0.252	351.34	0.923	0.921	0.995	2.341	0.061
356.10	0.172	0.533	2.583	1.055	0.208	352.05	0.659	0.731	1.077	1.741	0.238	351.37	0.942	0.940	0.994	2.359	0.044
355.55	0.199	0.547	2.339	1.082	0.232	352.00	0.689	0.746	1.053	1.806	0.220	351.38	0.950	0.948	0.993	2.371	0.037
354.95	0.230	0.563	2.130	1.112	0.256	351.85	0.720	0.764	1.038	1.876	0.203	351.40	0.967	0.965	0.993	2.417	0.022
354.55	0.275	0.577	1.854	1.161	0.278	351.70	0.767	0.796	1.021	1.962	0.173	351.43	0.986	0.984	0.991	2.603	0.005
354.10	0.337	0.598	1.595	1.229	0.294												
Ethanol (1) + water (2) at 300 kPa																	
399.0	0.032	0.228	4.309	1.004	0.051	386.0	0.334	0.569	1.510	1.236	0.279	382.9	0.695	0.742	1.040	1.800	0.207
394.0	0.073	0.364	3.479	1.010	0.100	385.6	0.363	0.581	1.436	1.273	0.285	382.7	0.726	0.761	1.028	1.870	0.191
391.4	0.109	0.422	2.916	1.037	0.149	385.2	0.402	0.598	1.351	1.319	0.287	382.5	0.772	0.790	1.009	0.990	0.164
390.1	0.140	0.460	2.572	1.047	0.172	384.7	0.447	0.617	1.273	1.383	0.287	382.3	0.823	0.829	1.000	2.105	0.131
388.9	0.176	0.488	2.250	1.078	0.204	384.2	0.501	0.640	1.196	1.465	0.280	382.2	0.863	0.865	0.998	2.157	0.103
387.8	0.221	0.516	1.959	1.117	0.235	383.8	0.555	0.666	1.138	1.546	0.265	382.1	0.925	0.923	0.996	2.261	0.057
387.2	0.255	0.533	1.786	1.150	0.252	383.2	0.637	0.708	1.073	1.693	0.236	382.1	0.944	0.942	0.996	2.283	0.042
Ethanol (1) + water (2) at 500 kPa																	
423.5	0.008	0.066	4.342	0.981	–0.007	404.4	0.264	0.523	1.700	1.163	0.251	400.2	0.641	0.710	1.065	1.656	0.222
420.1	0.021	0.145	3.948	0.997	0.026	403.9	0.312	0.547	1.524	1.200	0.257	399.2	0.729	0.761	1.032	1.868	0.193
417.2	0.034	0.222	4.010	0.996	0.044	402.9	0.346	0.563	1.455	1.255	0.278	398.9	0.780	0.796	1.018	1.985	0.164
413.3	0.067	0.328	3.318	0.995	0.076	402.5	0.404	0.589	1.317	1.312	0.273	398.8	0.801	0.811	1.012	2.041	0.152
410.7	0.098	0.385	2.848	1.015	0.116	401.6	0.445	0.607	1.264	1.385	0.285	398.7	0.850	0.853	1.006	2.117	0.117
408.1	0.144	0.444	2.394	1.044	0.162	401.2	0.513	0.640	1.168	1.466	0.266	398.6	0.902	0.901	1.004	2.195	0.080
406.5	0.183	0.475	2.104	1.083	0.201	400.9	0.565	0.668	1.116	1.529	0.246	398.9	0.947	0.944	0.993	2.280	0.037
406.0	0.203	0.487	1.971	1.101	0.214												
Ethanol (1) + water (2) at 700 kPa																	
435.60	0.012	0.074	3.402	0.998	0.013	415.20	0.329	0.549	1.494	1.240	0.276	410.30	0.776	0.795	1.038	1.964	0.180
429.70	0.042	0.241	3.613	0.982	0.037	413.10	0.470	0.617	1.240	1.418	0.286	410.30	0.802	0.812	1.026	2.039	0.162
424.90	0.081	0.340	2.959	1.011	0.098	412.30	0.537	0.648	1.163	1.529	0.278	410.30	0.831	0.834	1.017	2.112	0.140
422.60	0.109	0.385	2.632	1.035	0.136	411.00	0.651	0.710	1.087	1.739	0.247	410.20	0.874	0.873	1.014	2.179	0.110
421.30	0.123	0.407	2.545	1.050	0.158	410.70	0.716	0.753	1.055	1.840	0.212	410.20	0.907	0.904	1.012	2.236	0.085
419.90	0.148	0.436	2.345	1.069	0.183	410.50	0.740	0.769	1.048	1.893	0.201	410.20	0.930	0.926	1.011	2.293	0.068
418.60	0.175	0.461	2.166	1.094	0.209	410.40	0.754	0.779	1.045	1.920	0.193	410.30	0.957	0.953	1.008	2.368	0.045
416.40	0.237	0.502	1.841	1.163	0.260												

a The combined expanded uncertainties U­(k = 2) are U­(*p*) = 1 kPa, U­(*T*) = 0.2 K, U­(*x*_1_) = U­(*y*_1_)=0.003.

Table 4 shows the results for each fitting function (*FF*) (see eqs 3–5) for the ethanol (1) + water (2) system at different pressures in this work. Additionally, the *p*–*T*–*x*_1_–*y*_1_ data was verified by considering the isobaric data at 0.1 MPa from the literature^7,19,32–37^ for the ethanol/water system (see Figure 3), as all of these are considered thermodynamically consistent.^7,17,19^

**Figure 3:**
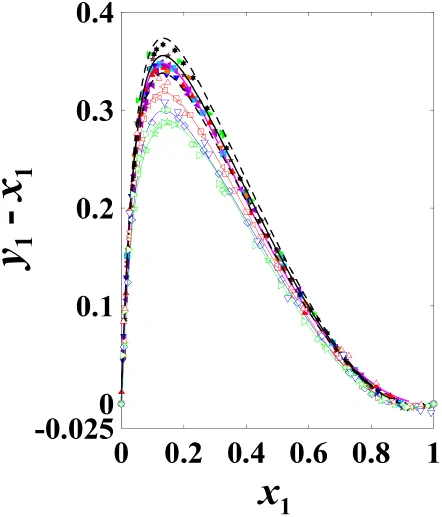
Experimental isobaric data of (y1 − x1) vs. x1 at 101 kPa for ethanol (1) + water (2) systems. This work (black solid six−pointed star) and the bibliographic data are by Otsuki and Williams^7^ (purple solid left–pointing triangle); by Kurihara et al.^32^ (green solid right–pointing triangle); by Carey and Lewis^33^ (blue solid square); by Kojima et al.^35^ (dark brown solid five–pointed star); by Kojima et al.^35^ (blue solid down–pointing triangle); by by Stabnikov et al.^36^ (pink solid curve); and by Stabnikov et al.^37^ (orange solid diamond). The isobaric data at other pressures are by Susial et al.^19^ at 100 kPa (red solid up–pointing triangle); this work at 300 kPa (red open square), at 500 kPa (blue open diamond), and at 700 kPa (green open five– pointed star); the bibliographic data by Otsuki and Williams^7^ (red open left–pointing triangle) at 344.7 kPa and (green open right– pointing triangle) at 689.5 kPa; and by Othmer et al.^18^ (red open up−pointing triangle) at 287.4 kPa, (blue open down–pointing triangle) at 515.0 kPa, and (green open six–pointed star) at 721.8 kPa. Fitting curves are at 101 kPa (black solid with dashed curves of 95% error), at 300 kPa (red solid), at 500 kPa (blue solid), and at 700 kPa (green solid).

Figure 3 shows the data set from this study and from the literature^7,19,32–37^ around 0.1 MPa. The correlation curves were obtained to fit all these data at 0.1 MPa together with the reliability curves for a 95% error margin. As can be seen, all the data, including that obtained in this study, lie within the error curves. Figure 3 also illustrates the data from this study at 300, 500, and 700 kPa, alongside the corresponding data from the literature^7,18^ under comparable isobaric conditions. In the latter case, only the data from Otsuki and Williams^7^ at 689.5 kPa show an equivalent trend to the data at 700 kPa obtained in this study. However, significant differences are observed for data at 689.5 kPa in the mole fraction range between 0.0 and 0.5, as illustrated in Figure 4. Figure 4 also more clearly illustrates the divergences between the data obtained in this study and the data from the literature^7,18^ under different isobaric conditions.

**Figure 4:**
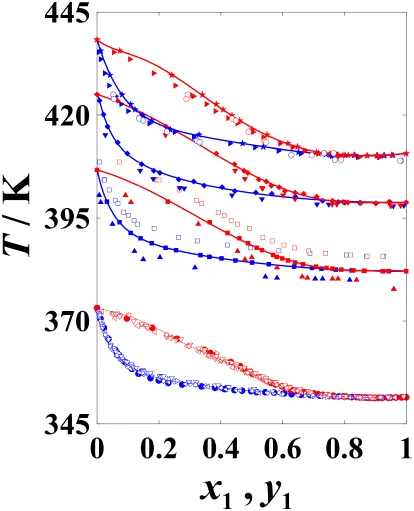
Experimental isobaric data of *T* vs. *x*_1_,*y*_1_ (blue and red colors) at 101 kPa for ethanol (1) + water (2) systems. This work (blue and red solid circles) and the bibliographic data are by Otsuki and Williams^7^ (blue and red open down–pointing triangles); by Kurihara et al.^32^ (blue and red open up–pointing triangles); by Carey and Lewis^33^ (blue and red open right–pointing triangles); by Kojima et al.^34^ (blue and red open diamonds); by Kojima et al.^35^ (blue and red open five–pointed stars); by Stabnikov et al.^36^ (blue and red solid curves); and by Stabnikov et al.^37^ (blue and red open six– pointed stars). The isobaric data at other pressures are by Susial et al.^19^ at 100 kPa (blue and red open left–pointing triangles); this work at 300 kPa (blue and red solid squares) with fitting curves, at 500 kPa (blue and red solid diamonds) with fitting curves, and at 700 kPa (blue and red solid five–pointed stars) with fitting curves; the bibliographic data by Otsuki and Williams^7^ (blue and red open squares) at 344.7 kPa and (blue and red solid right–pointing triangles) at 689.5 kPa; and by Othmer et al.^18^ (blue and red solid up–pointing triangles) at 287.4 kPa, (blue and red solid down–pointing triangles) at 515.0 kPa, and (blue and red open circles) at 721.8 kPa.

The *p*–*T*–*x*_1_–*y*_1_ thermodynamic treatment data were obtained using the γ–Φ approach and the subroutines presented in the FORTRAN program of Fredenslund et al.^24^

The activity coefficient of the liquid phase (γ) can be expressed for the binary mixture as follows, accepting that the fugacity of pure liquid at the system’s temperature and pressure is the standard state and considering isofugacity as the equilibrium condition:

6 $$\gamma_{i}=\frac{y_{i}p}{x_{i}p_{i}^{0}}\exp\left[\frac{p}{RT}\left(2\underset{j}{\sum }y_{i}B_{ij}-\underset{i}{\sum }\underset{j}{\sum }y_{i}y_{j}B_{ij}\right)-\frac{p_{i}^{0}B_{ii}}{RT}+\frac{\left(p_{i}^{0}-p\right)\times v_{i}^{L}}{RT}\right]$$

The fugacity coefficient (ϕ_*i*_) of the binary mixture was calculated by truncating the second term of the virial equation of state:

7 $$\phi_{i}=\phi_{i}^{0}\exp\left[\frac{p}{RT}\left(2\overset{2}{\underset{j=1}{\sum }}y_{i}B_{ij}-\overset{2}{\underset{i=1}{\sum }}\overset{2}{\underset{j=1}{\sum }}y_{i}y_{j}B_{ij}\right)-\frac{p_{i}^{0}B_{ii}}{RT}\right]$$

The properties used for VLE data processing in this study are presented in Table 3. The association parameters (ETA_*i*_) obtained in a previous work^15^ were used to calculate the virial coefficients (*B*_*ii*_, *B*_*ij*_) using the Hayden and O’Connell method.^38^ The solvation parameter (ETA_*ij*_) used was obtained from Fredenslund et al.‘s book.^24^ The molar volume of the pure liquid component (*v*_*i*_^L^) was estimated using the Yen and Woods equation.^39^ The critical properties used for the isobaric data treatment in this work were obtained from Daubert and Danner.^22^ The Antoine equation constants (see Table 3) were obtained from the vapor pressure data of pure substances (see Table 2). The results of the activity coefficients and the excess Gibbs function are shown in Table 5.

### 3.3 Azeotropic Data

In a previous paper, we used bibliographic data^19,20^ to correlate the azeotrope points of the ethanol and water mixture. To obtain the azeotrope data for the ethanol/water mixture, the Thomas algorithm adapted with the natural cubic spline method was used. The critical properties^22^ and the association and solvation parameters^24^ were applied. The data correlation results obtained were previously presented^19^ as follows:

8 $$x_{1,az}=\frac{0.818-1.531\times T_{R,az}}{1-1.841\times T_{R,az}}$$

9 $$\log_{10}p_{R,az}=\frac{11.451-11.648\times T_{R,az}}{1-4.734\times T_{R,az}}$$

To carry out the experimental determination of the azeotrope at 101 kPa in the multipurpose rectification tower, a sufficiently large quantity of an ethanol and water mixture with a known composition must first be prepared. This allows the volumetric quantities extracted from the equipment via the valves in sections {2} and {6} (see Figure 1) to be replaced by the loading section {3} (see Figure 1) during the test.

The distillation tower only has two small rectification sections. It takes several days to rectify the mixture and obtain the azeotrope data for the ethanol and water mixture. However, preparing mixtures of ethanol and water with different compositions can reduce working time. The two–phase composition must be monitored every 2 h to evaluate its evolution over time. In this study, the azeotrope was obtained by operating the rectification tower at total reflux. The azeotropic point obtained using the multipurpose rectification tower was the following:

$$x_{1,az}=0.899;\;T_{az}/K=351.3;{\;p}_{az}/kPa=101$$

It can be seen that the temperature and composition of the ethanol/water azeotrope are consistent with the values reported in the literature^19,20^ (see Figure 5). Nevertheless, to validate the experimental equipment, the properties referenced in the bibliography^22^ were used to calculate for the azeotrope the reduced temperature of the mixture as *T_R_*,*_mix_* = 0.6635 and the reduced pressure as log_10_ *P_R_*,*_ac_* = −1.8969. However, eqs 8 and 9 give *x*_1,*az*,*calc*_ = 0.8932 and log_10_ *P_R_*,_*az*,_ *_calc_* = −1.8917 Therefore, considering the correlation curves obtained in previous work,^19^ the error in the experimental data is 0.64% and 1.21% with respect to the composition and pressure of the azeotrope, respectively.

**Figure 5:**
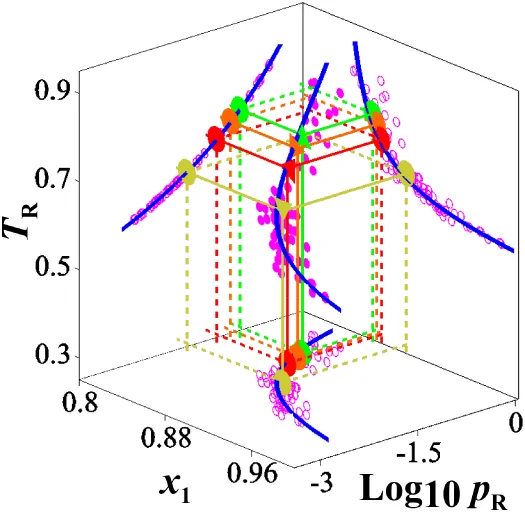
Three–dimensional plot of the bibliographic^19,20^ (magenta solid circle) and azeotropic points of this work at 101 kPa (banana yellow solid down–pointing triangle), 300 kPa (red solid left–pointing triangle), 500 kPa (orange solid right–pointing triangle) and 700 kPa (green solid up–pointing triangle) for the ethanol (1) + water (2) system with fitting curves blue lines from eqs 8 and 9. The symbols as solid circle from 101 kPa (banana yellow solid circle), 300 kPa (red solid circle), 500 kPa­(orange solid circle), 700 kPa (green solid circle) and bibliographic^19,20^ (magenta open circle) are for by–dimensional representation.

Furthermore, the azeotrope points corresponding to 300, 500, and 700 kPa were deduced using the correlations presented in Table 4. The results obtained, *x*_1,*az*_ = 0.876, *T*_*az*_/K = 382.2, *p*_*az*_/kPa = 300; *x*_1,*az*_ = 0.872, *T*_*az*_/K = 398.8, *p*_*az*_/kPa = 500 and *x*_1,*az*_ = 0.868, *T*_*az*_/K = 410.2, *p*_*az*_/kPa = 700 are shown in Figure 5 using the reduced coordinates. As can be seen, they are adequately coordinated with eqs 8 and 9.

### 3.4 Thermodynamic Verification of VLE Data

The thermodynamic consistency of the experimental data in this study was verified using three tests: the area test,^40,41^ the Wisniak test,^42^ and the point-to-point test by Fredenslund et al.^24^

Redlich and Kister^40^ proposed a method for verifying available experimental data. This method requires the activity coefficients calculated from the experimental data to be applied to the equations in Table 6. The areas below and above the curve are calculated by solving the integral of ln­(γ_1_/γ_2_) versus *x*_1_. For the *D* parameter to be used to calculate these areas, the condition must be less than 10%. The results presented in Table 6 for the ethanol + water system satisfy this condition for all pressures of this work.

**Table 6:** Verification Results for Ethanol (1) + Water (2) Binary Systems kPa

Redlich–Kister^40^ and Herington^41^ test					
*p*/kPa	$L=\int_{x}^{0}In\frac{\gamma_{1}}{\gamma_{2}}\partial x_{1}$	$W=\int_{l}^{x}In\frac{\gamma_{1}}{\gamma_{2}}\partial x_{1}$	$D=100\times |\frac{L-W}{L+W}|<10$	$J=150\times |\frac{T_{1}^{0}-T_{2}^{0}}{T_{\min}}|<10$	|D – J| < 10
101	0.32199	–0.31034	1.84	9.26	7.42
300	0.28746	–0.31579	4.70	9.62	4.92
500	0.27371	–0.31270	6.65	9.85	3.21
700	0.25931	–0.30426	7.98	10.09	2.12

Wisniak^42^ test				
*p*/kPa	$I_{1}=\int_{0}^{1}L_{i}\times \partial x_{1}$	$I_{2}=\int_{0}^{1}W_{i}\times \partial x_{1}$	$E=100\times |\frac{I_{1}-I_{2}}{I_{1}+I_{2}}|<5$	$\%\mathrm{Wisniak index:}\;0.95<\frac{L_{i}}{W_{i}}<1.08$
101	8.093	7.618	3.02	93.0
300	9.167	8.371	4.54	19.0
500	9.351	8.443	5.10	9.1
700	10.673	9.591	5.34	0.0

The Herington^41^ test checks overall thermodynamic consistency of the VLE data, while the Wisniak^42^ test simultaneously conducts point-to-point and area tests. According to the Herington^41^ method, isobaric VLE data are considered thermodynamically consistent if |*D* – *J*| < 10. In the Wisniak^42^ test, the system is considered thermodynamically consistent when the parameter *E* is less than 5. Additionally, the percentage of data that meets the criterion 0.9 < *L*_*i*_/*W*_*i*_ < 1.08 was evaluated to determine the proportion of data satisfying the Wisniak index. Table 6 shows that the ethanol + water system in this study passed the Herington^41^ test; however, the Wisniak^42^ test is only verified by the system at 101 kPa.

The Wisniak^42^ test is a function of vaporization enthalpies. The correlations reported in the literature^22^ were used to calculate the necessary enthalpies of vaporization. The activity coefficients were calculated on the basis of a non–ideal vapor phase. However, the Wisniak^42^ test is a function of vapor pressures and enthalpies of vaporization, and the latter can significantly affect and cause the results obtained.

The point-to-point method^24,43^ was also applied to the data in this study at 101, 300, 500, and 700 kPa using properties from the literature.^15,21–24^ The data from the literature^7,17,18,32–37^ used in Figures 3 and 4 were analyzed using the Fredenslund et al. test^24,43^ for verification, as well as bibliographic data.^25,44,45^ The results obtained are shown in Tables S1–S3 for isobaric systems and Tables S4–S6 for isothermal systems, both of which are included in the Supporting Information file.

Table S1 shows positive consistency in the VLE data at 101 and 300 kPa from this study when the point-to-point test^24^ is applied using properties from the literature^15,22^ and constants from the Antoine equation (see Table 3). These constants were obtained using the vapor pressures shown in Table 2. However, the data at 500 kPa are of questionable quality, and those at 700 kPa are inconsistent. This situation corresponds to that shown with respect to the data presented by Otsuki and Williams^7^ for different atmospheric pressures.

Given the influence of vapor pressures on the results obtained using the point-to-point test,^24^ it was deemed appropriate to analyze the data presented in Table S1 using different constants for the Antoine equation. Table S2 shows the results of analyzing the same systems using the Antoine constants from the literature.^22^ The same analysis was performed using the Antoine constants corresponding to reference,^23^ and the results are shown in Table S3. The same verification process was followed for the isothermal systems described in the literature^17,45^ (see Tables S4–S6).

The results obtained for the data in this study, as well as for data from Otsuki and Williams^7^ using different Antoine constants from refs 22,23 exhibit similar behavior to that mentioned above. However, this situation is also observable with data from other authors. See, for example, Tables S1–S6 for Álvarez et al.^25^ at 101.3 kPa, Van Zandijcke and Verhoeye at 101.3 kPa, Kurihara et al. at 333.15 K, and Yamamoto et al. at 298.15 K in Gmehling et al.^17^

This is why the effect of the Barker^46^ procedure, as used in the Fredenslund et al.^24^ routine, was evaluated. It is an optimization and minimization procedure applied to the integrated Gibbs–Duhem equation, which involves an additive function and a polynomial equation with a random number of coefficients.

## 4 The Fredenslund et al.^24^ Test vs PTDGE Routine Checking

Since sufficient excess enthalpy data^47^ exist for the ethanol/water system, we will apply this property to perform a test of points on the differential Gibbs equation (PTDGE) for verification of the VLE data on homogeneous binary mixtures of nonelectrolytes. This will allow us to analyze the effect that excess enthalpy might have when used in the validation procedure for systems at low and moderate pressures and temperatures as well as the enumerated situations mentioned in the Section 3.4 of the manuscript.

Furthermore, the Fredenslund et al.^24^ test, by using the Barker^46^ procedure, involves an iterative process and minimization of pressure deviation. Therefore, it is reasonable to assume that this circumstance could affect the validation of the data. On the other side, with the PTDGE procedure, an activity coefficient model is used instead of a polynomial equation; this is why applying the Gibbs differential equation directly will reveal the impact of these factors on the verification results.

To verify the systems used in this study, as well as those cited in Tables S1–S6, the properties and Antoine constants shown in Table 3 were employed. This was also done to verify the quality of the data obtained using the new multipurpose equipment and, consequently, to validate its operational quality.

Therefore, the autocorrelation process of the experimental errors must be verified. This can be observed by applying the FORTRAN routine developed by Fredenslund et al.,^24^ which enables the point errors to be redistributed across properties. It also generates valid data, which may not be the case otherwise. Additionally, the effect of excess enthalpy is incorporated into the PTDGE verification process. For these reasons, a procedure based on the fundamental relation for excess properties and using differential Gibbs excess energy is being considered:

10 $$\partial \left(\frac{g^{E}}{RT}\right)=\frac{v^{E}}{RT}\partial p-\frac{h^{E}}{RT^{2}}\partial T+\overset{n}{\underset{i=1}{\sum }}\ln\gamma_{i}\times \partial x_{i}$$

Equation 10 can be applied to a binary system under isobaric or isothermal conditions, together with the expression for the excess Gibbs function and activity coefficients, which postulates the activity coefficient model of the NRTL:^48^

11 $$\frac{g^{E}}{RT}=x_{i}\times \left(1-x_{i}\right)\times \left[\frac{\frac{\Delta_{21}}{RT}\times \exp\left(-\frac{\alpha \times \Delta_{21}}{RT}\right)}{x_{1}+\left(1-x_{1}\right)\times \exp\left(-\frac{\alpha \times \Delta_{21}}{RT}\right)}+\frac{\frac{\Delta_{12}}{RT}\times \exp\left(-\frac{\alpha \times \Delta_{12}}{RT}\right)}{\left(1-x_{1}\right)+x_{1}\times \exp\left(-\frac{\alpha \times \Delta_{12}}{RT}\right)}\right]$$

Considering eq 10, the following expression for the activity coefficients can be deduced:

12 $$\ln\gamma_{i}=\frac{g^{E}}{RT}\left(1-x_{i}\right)\times \left[\frac{\partial \left(\frac{g^{E}}{RT}\right)}{\partial x_{i}}+\left(\frac{h^{E}}{RT^{2}}\right)\times \frac{\partial T}{\partial x_{i}}-\left(\frac{v^{E}}{RT}\right)\times \frac{\partial p}{\partial x_{i}}\right]$$

Furthermore, after the selected activity coefficient model^48^ has been considered, the following expression for the activity coefficients must be applied to eq 10:

13 $$\begin{array}{l} \frac{\partial \left(\frac{g^{E}}{RT}\right)}{\partial x_{1}}=\left(1-2x_{1}\right)\times \left[\frac{\frac{\Delta_{21}}{RT}\times \exp\left(-\frac{\alpha \times \Delta_{21}}{RT}\right)}{x_{1}+x_{2}\times \exp\left(-\frac{\alpha \times \Delta_{21}}{RT}\right)}+\frac{\frac{\Delta_{12}}{RT}\times \exp\left(-\frac{\alpha \times \Delta_{12}}{RT}\right)}{x_{2}+x_{1}\times \exp\left(-\frac{\alpha \times \Delta_{12}}{RT}\right)}\right]-\\ -x_{1}x_{2}\times \left\{\frac{\frac{\Delta_{21}}{RT}\times \exp\left(-\frac{\alpha \times \Delta_{21}}{RT}\right)\times \left[1-\exp\left(-\frac{\alpha \times \Delta_{21}}{RT}\right)\right]}{{[x_{1}+x_{2}\times \exp\left(-\frac{\alpha \times \Delta_{21}}{RT}\right)]}^{2}}+\frac{\frac{\Delta_{12}}{RT}\times \exp\left(-\frac{\alpha \times \Delta_{12}}{RT}\right)\times \left[\exp\left(-\frac{\alpha \times \Delta_{12}}{RT}\right)-1\right]}{{[x_{2}+x_{1}\times \exp\left(-\frac{\alpha \times \Delta_{12}}{RT}\right)]}^{2}}\right\} \end{array}$$

In the absence of experimental data, the excess enthalpy can be calculated using the following formula:

14 $$\frac{h^{E}}{RT^{2}}=-{[\frac{\partial \left(g^{E}/RT\right)}{\partial T}]}_{P,x}$$

As the application of eq 14 requires the differential of the excess Gibbs energy with respect to temperature, the following equation,

15 $$\begin{array}{l} \frac{\partial \left(\frac{g^{E}}{RT}\right)}{\partial T}=-\frac{1}{T}\times \frac{g^{E}}{RT}+\frac{x_{1}\times x_{2}}{T}\times \frac{\alpha \times {\left(\frac{\Delta_{21}}{RT}\right)}^{2}\times \exp\left(-\frac{\alpha \times \Delta_{21}}{RT}\right)}{x_{1}+x_{2}\times \exp\left(-\frac{\alpha \times \Delta_{21}}{RT}\right)}\left(1-\frac{x_{2}\times \exp\left(-\frac{\alpha \times \Delta_{21}}{RT}\right)}{x_{1}+x_{2}\times \exp\left(-\frac{\alpha \times \Delta_{21}}{RT}\right)}\right)+\\ +\frac{x_{1}\times x_{2}}{T}\times \frac{\alpha \times {\left(\frac{\Delta_{12}}{RT}\right)}^{2}\times \exp\left(-\frac{\alpha \times \Delta_{12}}{RT}\right)}{x_{2}+x_{1}\times \exp\left(-\frac{\alpha \times \Delta_{12}}{RT}\right)}\left(1-\frac{x_{1}\times \exp\left(-\frac{\alpha \times \Delta_{12}}{RT}\right)}{x_{2}+x_{1}\times \exp\left(-\frac{\alpha \times \Delta_{12}}{RT}\right)}\right) \end{array}$$

is necessary when the selected activity coefficient model^48^ is applied.

For the systems in this study, the data reported by Larkin^47^ can be used to calculate the enthalpy of excess using eq 10. We correlated this data to a Redlich–Kister-type equation as follows:

16 $$h^{E}=x_{1}\times \left(1‐x_{1}\right)\times \overset{n}{\underset{i=1}{\sum }}\left(a_{i}+b_{i}\times T\right)\times {\left(2+x_{1}‐1\right)}^{i‐1}$$

The results of correlating the data presented by Larkin^47^ for molar enthalpy of excess to eq 16 are as follows:


*a*_1_

*a*_2_

*a*_3_

*a*_4_

*a*_5_
–15781.786711.99–10971.418878.91–5650.53


*b*_1_

*b*_2_

*b*_3_

*b*_4_

*b*_5_
47.4534–17.305727.8475–21.530711.8449

The process of applying the point test to the differential Gibbs excess equation (PTDGE), as developed in this work on the ethanol and water mixtures, is based on the application of eqs 6 and 7 together with eqs 10 to 16, as well as the Antoine equation (eq 1). The steps are

Considering the Hayden–O’Connell^38^ procedure, the Yen–Woods^39^ equation, critical properties from the literature,^22^ and Antoine constants (see Table 3), the following properties are calculated: vapor pressure $\left(p_{i}^{0}\right)$ , saturated liquid volume $\left(v_{i}^{L}\right)$ , reference fugacity coefficient $\left(\phi_{i}^{0}\right)$ , fugacity coefficient of the vapor phase (*ϕ_i_*), and liquid phase activity coefficient (*γ_i_*).
To verify the system, the excess molar enthalpy is calculated for each *T* – *x*_1_ pair using eq 16 and the constants obtained in this study. This is done by employing the data reported by Larkin^47^ to obtain the value of the term (*h^E^*/*RT* ^2^).
A Redlich–Kister-type polynomial equation can be used to correlate pairs of *T* vs *x*_1_ values. The derivative of the obtained correlation is determined, and the derivative value (*∂T*/*∂x*_1_) is calculated for each point.
Equation 10 is applied, and the value of the term [*∂*(*g^E^*/*RT*)/*∂x*_1_]_exp_ is obtained for each point. For isothermal systems, the quantities corresponding to the excess volume and excess enthalpy can be considered zero. For isobaric systems, it is estimated that the quantity corresponding to the excess volume is zero. For systems where excess enthalpy is unavailable and cannot be assumed to be zero, follow the procedure indicated previously and apply using eq 14.
Equation 13 is obtained using a selected activity coefficient model.^48^ The pairs of values *x*_1_ and [*∂*(*g* ^E^ */RT)/∂x*_1_]_exp_ are correlated with respect to eq 13. For ethanol/water systems, *α* = 0.3 is assumed. Using eq 12 and the constants obtained through correlation functions, (In *γ*_1,_ *_calc_*) and (In *γ*_2,_ *_calc_*) are calculated. The simplifications mentioned in Step 4 are also considered.
To obtain the calculated pressure (*p*_*calc*_), the functions (In *γ*_1,calc_) and (In *γ*_2,calc_) are used in the following equation:
17 $$p_{calc}=\frac{\gamma_{1,calc}\times x_{1}\times p_{1}^{0}\times \varphi_{1}^{0}}{\phi_{1}}\left[\exp\frac{\left(p_{1}^{0}-p\right)v_{1}^{L}}{RT}\right]+\frac{\gamma_{2,calc}\times x_{2}\times p_{2}^{0}\times \varphi_{2}^{0}}{\phi_{2}}\left[\exp\frac{\left(p_{2}^{0}-p\right)v_{2}^{L}}{RT}\right]$$
To determine, (*y_i_*,*_calc_*) the previously calculated properties (*p_calc_*) and (In *γ_i_*,*_calc_*) are used, along with the corresponding equilibrium condition eq (eqs 6 and 7) when using the *γ–ϕ* approximation.
18 $$x_{i}\times \gamma_{i,calc}\times p_{1}^{0}\times \phi_{i}^{0}=y_{i}\times p_{calc}\times \phi_{i}^{V}\times \exp\left[\frac{v_{i}^{L}\left(p_{1}^{0}-p_{calc}\right)}{RT}\right]$$

This allows us to evaluate each specific difference in the data between the experimental (*p*, *yi*) and calculated properties (*p_calc_*, *y_i_*,*_calc_*). For this purpose, the reliability limit for the calculated data was set at 1.5% of the experimental pressure value, while for the vapor phase composition, 1% of the difference between the experimental and calculated values was established. These limits are similar to those established by Fredenslund et al.^24^

To ensure the consistency of the evaluated system data, it was established that the number of points meeting the reliability conditions for both pressure and composition had to exceed 75% of the total N of the evaluated data. Although this assignment was randomly established, it seems statistically reasonable. Therefore, it has been proceeded similarly to how other authors^24,40–42^ treated the limiting assignments of their validation procedures.

Based on the Gibbs–Duhem differential equation, a decision algorithm was prepared for the procedure described above, as shown in Figure 6. This algorithm was applied to data from the literature,^7,17,19,25,44,45^ as well as to data from this study. The thermodynamic properties from the literature^15,22,24^ and the Antoine constants presented in Table 3 were also used. The results are presented in Tables S1 to S6.

**Figure 6:**
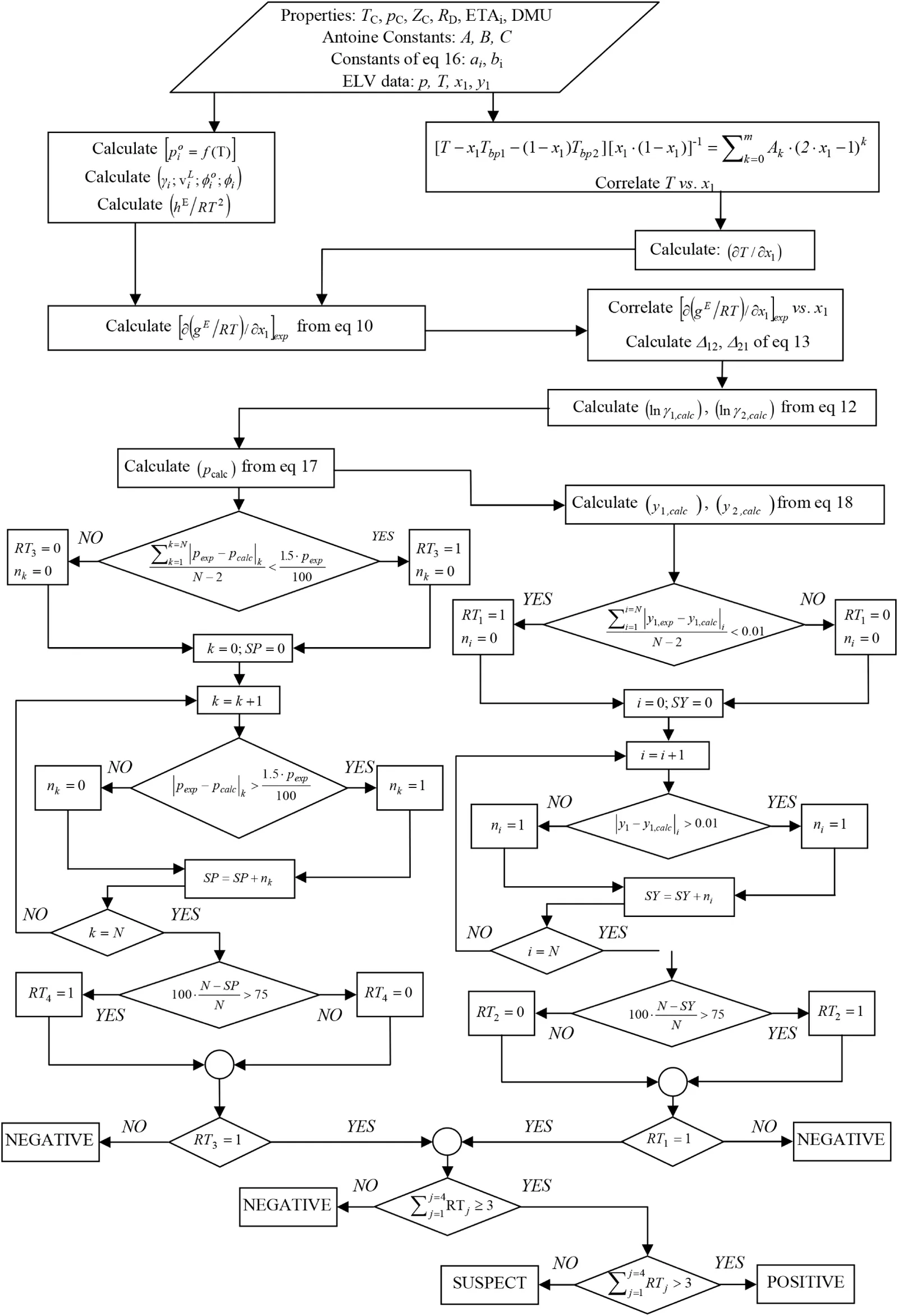
Decision diagram for the proposed PTDGE procedure.

The Fredenslund et al.^24^ test was applied with the same properties to verify the effect of vapor pressures and analyze discrepancies with respect to the PTDGE procedure, as shown in Tables S1 to S6.

The PTDGE program uses the Nelder and Mead^49^ algorithm because the simplex method of nonlinear programming is commonly used in chemical engineering. The PTDGE procedure implemented in FORTRAN differs from that described by Fredenslund et al.^24^ in that it replaces the GAUSL subroutine by a NELDER subroutine. In this NELDER subroutine the Nelder and Mead^49^ algorithm has been programmed in FORTRAN. The GAUSL subroutine solves a group of linear algebraic equations using Gaussian elimination with row pivoting. In contrast, the NELDER subroutine uses a numerical method to optimize unconstrained objective functions.

Another difference lies in the direct use of the Gibbs differential equation (eq 10) to obtain the NRTL^48^ model parameters in the PTDGE procedure. After obtaining these parameters, the properties can be directly determined using eq 12. This enables the pressure at each point to be calculated using eq 17. However, the FORTRAN routine developed by Fredenslund et al.^24^ uses Legendre polynomial equations and their derivatives to express the Gibbs excess energy function in eq 17 to correlate the experimental data until a minimum pressure deviation value is obtained.

The PTDGE procedure incorporates the calculation of excess enthalpy using either eq 14 or, for the ethanol+water system, applying eq 16. Furthermore, the PTDGE test employs the NRTL,^48^ an activity coefficient model, to replace the correlation of activity coefficients, which in the point-to-point^24^ test is performed using Legendre polynomials.

A first approximation was initially made in the study of the systems. The results are shown under the PTDGE columns in Tables S1 to S3. This is possible because the systems presented were obtained at low pressure; therefore, it is an adequate approximation to consider that the excess enthalpy in eq 10 can be simplified. Operating in this manner allows the systems to be evaluated using a factored function for the activity coefficients. In contrast, the point-to-point test^24^ procedure uses an additive function. This allows us to quantify the effect that arises during data validation when minimizing errors in the correlation function.

A total of 106 isothermal and isobaric systems of the ethanol and water mixture were analyzed and verified. Fifty-three isobaric systems are presented in Tables S1 to S3, and twenty-seven isothermal systems are included in Tables S4 to S6. The systems obtained in this study at 100, 300, 500, and 700 kPa are also included in Tables S1 to S3. Properties from the literature^15,22,24^ and vapor pressures from this work and the literature^22,23^ were used in all cases. To correlate the data, the objective function (*OF*1),

19 $$OF1={[{\left(\ln\frac{\gamma_{1}}{\gamma_{2}}\right)}_{\exp}-{[\frac{\partial \left(g_{m}^{E}/RT\right)}{\partial x_{1}}]}_{calc}]}^{2}$$

was used as the statistical parameter for the regression. The results of the evaluated systems corresponding to the ethanol/water mixture are presented in Tables S1 to S6.

The positive results obtained from the verification of isobaric systems, considering the Fredenslund et al. and PTDGE tests, respectively, were 50.9% and 45.3%, applying the vapor pressures of this work; 43.4% and 18.9%, applying the vapor pressures of Daubert and Danner;^22^ and 60.4% and 37.7%, applying the vapor pressures of VDI.^23^

The positive results obtained from isothermal system validation using the Fredenslund et al. and PTDGE tests, respectively, were 7.4% and 7.4%, considering the vapor pressures of this work; 25.9% and 0%, considering the vapor pressures of Daubert and Danner,^22^ and 25.9% and 18.5%, considering the vapor pressures of VDI.^23^

Systems that were deemed suspicious were not considered positive. The boiling point pressures of the pure components reported by various authors under isothermal conditions were disregarded because, in many cases, these pressures and their corresponding error ranges did not align well with those deduced from the bibliographic Antoine constants^22,23^ for the system’s temperature.

As shown in Tables S1 and S6, the results indicate that analyzing systems with different Antoine constants is preferable for a more accurate description of system quality. A higher degree of rigor is observed when assessing system quality using the PTDGE routine. Therefore, it can be concluded that the PTDGE procedure, which uses the differential Gibbs excess function, is much more restrictive than the Fredenslund et al.^24^ method.

### 4.1 Analysis of the Results

When conducting a comparative analysis of the results presented in Tables S1–S6, we employed an approximation for isobaric systems, whereby the excess enthalpy term in eq 10 is estimated as zero. The results obtained using this approximation are shown in the PTDGE column of Tables S1–S3. These results were analyzed in relation to those presented in the Fredenslund et al.^24^ column. Similarly, the columns with the same designation in Tables S4–S6 are analyzed for isothermal systems.

Significant differences are observed in the results shown in Tables S1–S6 obtained using different vapor pressures. When performing the analysis, we considered results that contradicted each other; therefore, we included results corresponding to systems showing positive consistency using the point-to-point test^24^ and negative consistency when applying the PTDGE procedure, and vice versa. Operating in this manner, and disregarding discrepancies in doubtful results, enables us to significantly verify the different extreme results. Furthermore, we selected systems exhibiting identical behavior when applying at least two of the vapor pressures used. Considering the results for the vapor pressures shown in Table 3, with respect to Tables S1 to S3, we observe that the systems corresponding to rows 4, 20, and 35 exhibit negative consistency when using the point-to-point test^24^ and positive consistency when applying the PTDGE procedure. Conversely, the systems in Tables S1 to S3, rows 8, 33, 37, and 44, and in Tables S4–S6, rows 13 and 14, exhibit positive consistency when using the point-to-point test^24^ and negative consistency when applying the PTDGE procedure.

After applying the point-to-point^24^ test and PTDGE procedure for the systems presented in Tables S1 to S3 (rows 35 and 44) and the isothermal system in Tables S4 to S6 (row 14), the results of the deviation for the properties are plotted in Figures 7 to 9.

**Figure 7:**
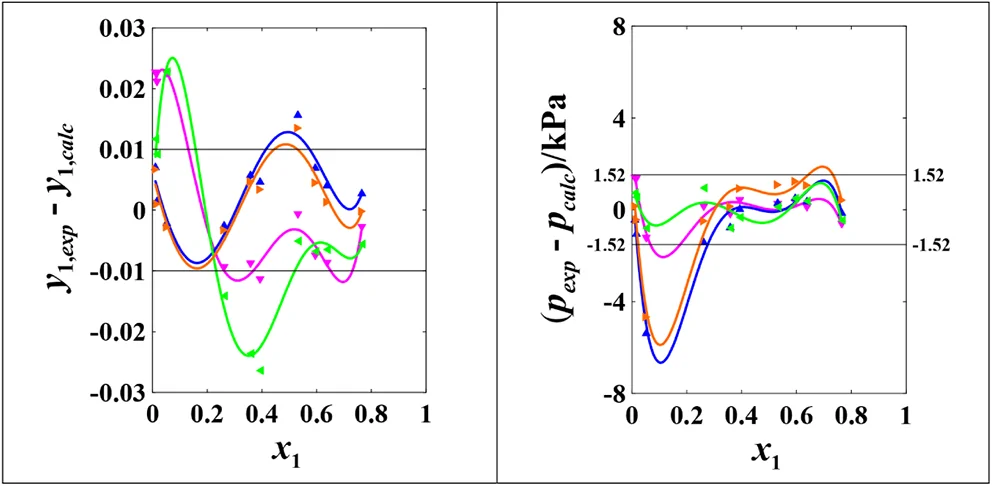
Representation of *y*_1,exp_ – *y*_1,calc_ vs. *x*_1_ and *p*_exp_ – *p*_calc_ vs. *x*_1_ deviation results obtained from thye isobaric system at 101.3 kPa of Chen et al. in Gmehling, J. et al.^17^ vol 1, part 1C, page 179. The results were obtained using the Fredenslund et al.^24^ test {magenta solid down–pointing triangle and green solid left–pointing triangle} and the PTDGE procedure {blue solid up–pointing triangle and orange solid right–pointing triangle}. The vapor pressures employed were obtained from this work of Table 3 (magenta solid down–pointing triangle and blue solid up–pointing triangle) and bibliographic^23^ data (green solid left–pointing triangle and orange solid right–pointing triangle).

As an example of systems that are negatively evaluated by the point-to-point test^24^ and positively by the PTDGE test, we analyzed the data from Chen et al. (see ref 17). Figure 7 shows that the point-to-point test^24^ rearranges the pressures ((see {magenta solid down–pointing triangle and green solid left–pointing triangle} in Figure 7) within the error interval, regardless of the vapor pressures used in the evaluation.

The FORTRAN program,^24^ by minimizing the pressure error at each point, redistributes and transfers the reduced pressure errors onto the vapor-phase compositions. This results in a significant increase in the deviations of the vapor-phase compositions, consequently rendering the system inconsistent when this test is applied.

Conversely, the PTDGE test shows (see {blue solid up–pointing triangle and orange solid right–pointing triangle} in Figure 7) that the data, corresponding to a composition of 0.0522 regardless of the vapor pressures used, are the source of the corrections applied to the errors by the point-to-point test.^24^ Therefore, since the PTDGE test does not relocate errors either globally or individually, it reports a system that globally satisfies the established criteria for property error. Consequently, the PTDGE test has confirmed that the system satisfies the consistency criteria and also reports a data point that should be eliminated because it exceeds the permissible error by more than three times.

Tables S1–S3 show isobaric systems that Fredenslund et al.^24^ report as having positive consistency but that the PTDGE test shows to be inconsistent. This case corresponds to the system in Tables S1–S3, row 44, reported by A.M. Kraehenbuehl (see ref 17). After applying the Fredenslund et al.^24^ test and PTDGE procedure of this work to previous data, deviation results obtained are shown in Figure 8. Deviation results with respect to pressure indicate that with the PTDGE test, only two data points, corresponding to mole fractions of 0.0493 and 0.09681, show a greater deviation from the maximum established when using the vapor pressures from the literature.^22,23^ Therefore, the set of all data satisfies the established criterion for the maximum pressure deviation. However, the deviations in the compositions reported with the PTDGE test indicate that the system is inconsistent.

**Figure 8:**
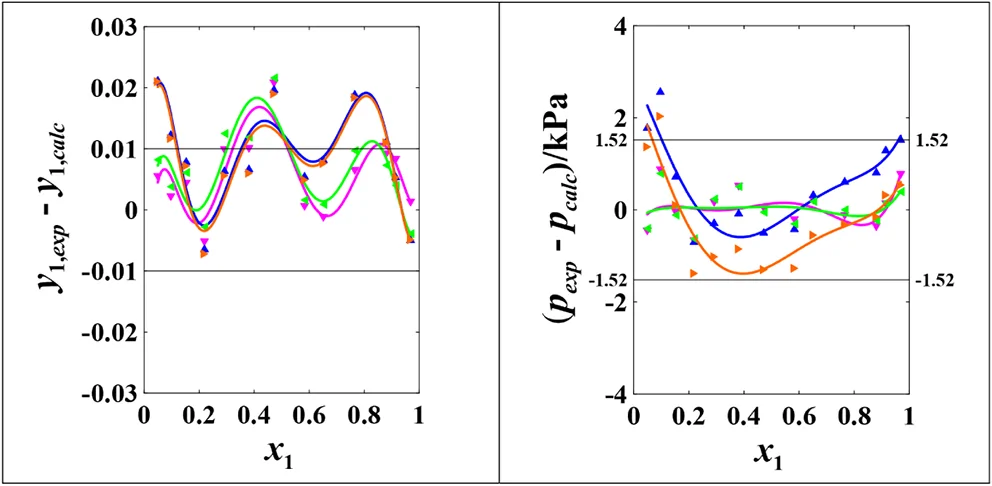
Plot of *y*_1,exp_ – *y*_1,calc_ vs *x*_1_ and *p*_exp_ – *p*_calc_ vs *x*_1_ deviation results for the isobaric system at 101.3 kPa of Kraehenbuehl, M.A. in Gmehling, J. et al.^17^ vol 1, part 1C, page 216. Data obtained considering the Fredenslund et al.^24^ test {magenta solid down– pointing triangle and green solid left–pointing triangle} and the PTDGE procedure {blue solid up– pointing triangle and orange solid right–pointing triangle}. The vapor pressures employed were from bibliographic^22^ data (magenta solid down–pointing triangle and blue solid up–pointing triangle) and bibliographic^23^ data (green solid left–pointing triangle and orange solid right–pointing triangle).

On the contrary, the reorganization that occurs in the pressure deviations (see {magenta solid down–pointing triangle and green solid left–pointing triangle} inFigure [Fig fig8]) in the vicinity of the axis of abscissas, performed by the point-to-point test,^24^ causes the deviations in composition to be globally relocated within the maximum error allowed for the deviation of compositions, although 3 data points are observed that do not satisfy the established criterion.

Different isothermal systems were also analyzed (see Tables S4–S6) using the FORTRAN test^24^ and the PTDGE procedure of this work. The experimental vapor pressures reported for the pure components by various authors in the literature^17^ were not considered. This is because many of them do not correlate well with the vapor pressures obtained by applying eq 1 and using the constants reported in Table 3.

Therefore, the vapor pressures required for the pure components when using the FORTRAN test^24^ were obtained directly from the Antoine constants reported in Table 3. Furthermore, considering this condition, both procedures for verifying the VLE data, the point-to-point test and the PTDGE procedure of this work, used the same reference condition for the thermodynamic treatment of the data.

The Antoine constants used to calculate the vapor pressures to analyze the isothermal system reported by Phutela et al. (see ref 17) are shown in Table 3. The results obtained (see Figure 9) when verifying the isothermal system reported by Phutela et al.^17^ using the vapor pressures from the literature^21–23^ (see {magenta solid down– pointing triangle and blue solid up–pointing triangle} with ref 21, see {green solid left–pointing triangle and orange solid right–pointing triangle} with ref 22, and see {dark brown solid square and red solid five–pointed star} with ref 23) indicate that the system is inconsistent with all of them considering the PTDGE test (see {blue solid up–pointing triangle, orange solid right–pointing triangle and red solid five–pointed star} in). Conversely, the distribution and minimization of the errors corresponding to each pressure as shown in Figure 9 (see {magenta solid down–pointing triangle, green solid left–pointing triangle and dark brown solid square}) indicate positive consistency when applying the FORTRAN test.^24^

**Figure 9:**
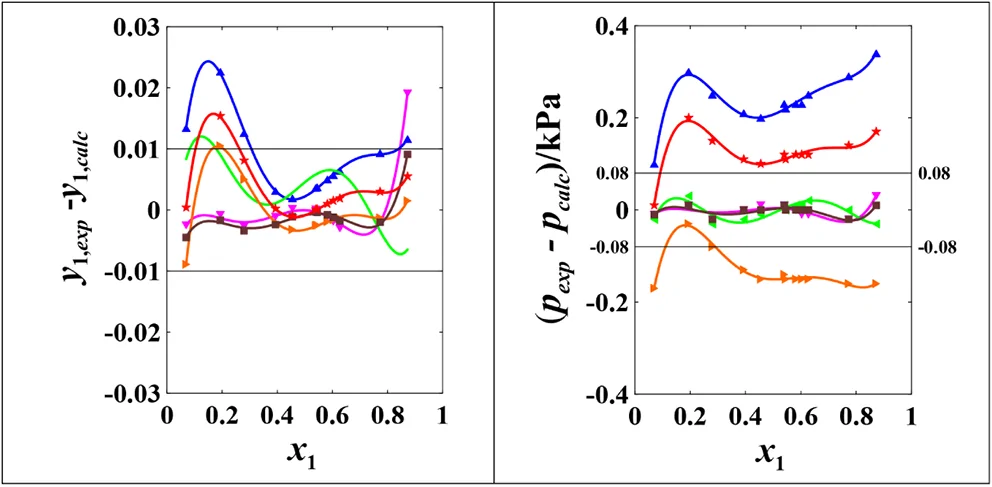
Deviations on *y*_1,exp_ – *y*_1,calc_ vs *x*_1_ and *p*_exp_ – *p*_calc_ vs *x*_1_ results for the isothermal system at 298.14 K of Phutela et al. in Gmehling, J. et al.^17^ vol 1, part 1B, page 108. Data obtained considering the point–to–point test of Fredenslund et al.^24^ {dark brown solid square, magenta solid down–pointing triangle and green solid left–pointing triangle} and the PTDGE procedure {red solid five–pointed star, blue solid up– pointing triangle and orange solid right–pointing triangle} using the vapor pressures from bibliography: ref 21 (dark brown solid square and red solid five–pointed star), ref 22 (magenta solid down–pointing triangle and blue solid up–pointing triangle) and ref 23 (green solid left–pointing triangle and orange solid right–pointing triangle).

Figure 9 shows the dispersion curves of the errors (see {blue solid up– pointing triangle, orange solid right–pointing triangle and red solid five–pointed star}) obtained for each vapor pressure. Comparing the different curves of the deviations reveals the effect of nonuniform random propagation of the errors generated by each vapor pressure. Therefore, to verify the VLE data, it may be advisable to analyze the systems applying different vapor pressures. This will allow for a better thermodynamic evaluation.

The analysis shows that the verification process developed by Fredenslund et al.^24^ modifies the uncertainty of the individual activity coefficients when they are grouped into the Gibbs excess function. This is a consequence of applying an additive function without weight factors. Therefore, the correlation of the Gibbs excess function is minimized with respect to the activity coefficient with the least uncertainty. This operation generates the overcorrelation of one of the properties and the undercorrelation of others.

The PTDGE procedure uses the differential of the Gibbs excess function directly (i.e., eq 10). Therefore, the effect that individual activity coefficients have on the Gibbs excess function is not modified. This is particularly evident when using the factored function, which is appropriate given that the excess enthalpy can be neglected in the isobaric case.

Furthermore, this new procedure does not redistribute or reallocate the error of the properties based on successive iterations, as occurs when correlating data using Barker’s procedure.^46^ In the PTDGE procedure, this is possible because the (*g^E^*/*RT*) function is estimated directly using its differential with respect to the composition. The (*g^E^*/*RT*) function is established invariably because the properties are not minimized. The properties are correlated by checking the minimum objective function of the correlation. Therefore, the pressure and vapor phase composition are calculated directly.

### 4.2 Application of Excess Enthalpy in the PTDGE Routine

As mentioned in the previous section, there is available experimental data^47^ for the excess enthalpy of the ethanol + water mixture. Therefore, it is possible to apply eq 2 without considering the corresponding simplification of this property.

The appropriate modification has been made to the FORTRAN program of the PTDGE procedure to include this property. For this purpose, we used eq 16 and the correlation constants shown in Section 4 of the manuscript. These constants were obtained for this work using data from the literature.^47^

Considering the excess enthalpy term in eq 10 for isobaric systems, in addition to the activity coefficients calculated from experimental data (see Section 3.2 of the manuscript), requires using eqs 13 and 14 for data correlation. For eq 14, we will use the data reported by Larkin;^47^ therefore, eq 16 will be applied.

Based on the above, the initial correlation of data to a factored function has transformed into an equation with several terms. In these cases, the maximum likelihood principle can be applied, which necessitates the consideration of property uncertainties.

Considering the property uncertainties reported in this work and the corresponding excess enthalpy uncertainty value taken from Larkin’s work,^47^ we calculated the propagation of uncertainties over each term of eq 10 for the isobaric case. The resulting standard deviation has been:

$$SD\left[\ln\left(\frac{\gamma_{1}}{\gamma_{2}}\right)\right]={8.1\times 10}^{-2};,SD\left[\frac{\partial \left(g^{E}/RT\right)}{\partial x_{1}}\right]={8.1\times 10}^{-2};,SD\left[\left(\frac{h^{E}}{RT^{2}}\right)\times \left(\frac{\partial T}{\partial x_{1}}\right)\right]={1.3\times 10}^{-4}$$

On the other hand, the weighted sum method can be applied to optimize a multiobjective function when Stadler’s^50^ optimization concept is considered. Thus, a multiobjective function can be obtained by multiplying a weighting factor by each individual objective function and adding the results.

Based on the obtained results, the minimum objective function^51^ can be established. In this function, the second term on the right-hand side of eq 10 must have a weighting factor of ***W*** = 1.6·10^–3^, while the weighting factor will equal one in the other two terms of the equation. Considering this, a FORTRAN program was prepared to verify isobaric systems using the correlation of the excess enthalpy expressed in eq 16 and the aforementioned weighting factor. The modified objective function (*OF*2) was then used to perform the data correlation.

20 $$OF2={\{{\left(\ln\frac{\gamma_{1}}{\gamma_{2}}\right)}_{\exp}-{[\frac{\partial (g^{E}/RT)}{\partial x_{1}}-\left(\frac{h^{E}}{RT^{2}}\right)\times \left(\frac{\partial T}{\partial x_{1}}\right)\times W]}_{calc}\}}^{2}$$

Applying this equation incorporated the new circumstances for data regression and for recalculating the properties pcalc and ycalc due to backsliding. The results are presented under the columns $\mathrm{PTDGE‐}h_{EXP}^{E}$ in Tables S1–S3.

The positive results obtained from the verification of isobaric systems, considering the $\mathrm{PTDGE‐}h_{EXP}^{E}$ tests, were 37.7%, applying the vapor pressures of this work; 28.3%, applying the vapor pressures of Daubert and Danner;^22^ and 43.4%, applying the vapor pressures of VDI.^23^ Systems that were deemed suspicious were not considered positive. The results indicate that analyzing systems with different Antoine constants is a suitable option.

Furthermore, as previously mentioned, the results obtained using the verification routine, in which excess enthalpy did not affect the data correlation (although this effect was subsequently corrected in the backsliding recalculation of the properties), are shown under the PTDGE column in Tables S1–S3.

When the systems deemed suspicious were considered consistent, the positive results obtained from verifying isobaric systems were 54.7% and 54.7% when applying the vapor pressures of this work, 45.3% and 41.5% when applying the vapor pressures of Daubert and Danner,^22^ and 58.5% and 54.7% when applying the vapor pressures of VDI,^23^ respectively, when considering the $\mathrm{PTDGE‐}h_{EXP}^{E}$ and PTDGE tests. Therefore, the results appear similar, suggesting that the enthalpy of evaporation’s effect on the tests may be insignificant.

When comparing the results obtained between the $\mathrm{PTDGE‐}h_{EXP}^{E}$ and PTDGE columns in Tables S1–S3 for the 159 tests performed, an average of 20% showed differences when the systems that were deemed suspicious were not considered positive. The number of cases in which differences in the verification results appeared was a function of the vapor pressures used. This suggests, as previously mentioned, that vapor pressures, as well as the propagation of errors when using eq 10, and therefore when including excess enthalpy in the verification of isobaric systems, have a significant effect on the final thermodynamic validation results of the data.

Furthermore, for comparative purposes, the PTDGE routine was prepared so that excess enthalpy could be calculated using eq 15, and by substituting it in eqs 14 and 10 could be used. In this way, the procedure was identical to that used with the experimental data. By verifying the systems presented in Tables S1–S3, considering the same weighting factors as those mentioned above, it was found that the numerical results did not show significant differences while the validation results turned out to be identical.

## 5 Conclusions

We present and describe a new multipurpose device for determining the vapor–liquid equilibria (VLE). It can be used to determine azeotropic points, as well as the vapor pressures of substances. The VLE data for the ethanol–water mixture were obtained at pressures of 101, 300, 500, and 700 kPa to study the evolution of the azeotrope and verify the device’s functionality.

The obtained VLE data were verified using standard thermodynamic consistency tests, including the Fredenslund et al. point-to-point test.^24^ Experimental data of this study were analyzed using different vapor pressures and showed positive consistency.

A new procedure (PTDGE) for verifying VLE data that uses a thermodynamic activity coefficient model is presented. In this study, the NRTL model was selected. This new procedure was analyzed and evaluated, and its use was justified by studying a significant number of ethanol–water systems at different pressures. The procedure directly applies the Gibbs–Duhem differential equation. It does not use correlation techniques to minimize property errors or perform iterative recalculations.

A decision diagram is used to describe the PTDGE procedure routine for its application. The PTDGE procedure applies the experimental excess enthalpies of the mixture.^47^ The application of the Gibbs excess energy differential with respect to temperature is analyzed to verify its use in the new VLE verification routine.

## Supplementary Material



## Notes

The results of the thermodynamic verification of ethanol/water systems obtained in this study and of various ethanol/water mixture systems from different bibliographic sources, as well as the results of applying different consistency tests to these systems. Thermodynamic validation was performed using the Fredenslund et al. test and the PTDGE procedure. This PTDGE procedure applied the data directly to the Gibbs differential equation. Tables S1–S3 include a comparison of the new PTDGE procedure with and without excess enthalpy applied to the differential Gibbs equation, as well as a comparison with Fredenslund et al.‘s test. The results of the consistency tests from isobaric systems are detailed in Tables S1–S3, and the results from isothermal systems are included in Tables S4–S6 (PDF)

## Nomenclature

*A,B,C*: Antoine constants
*DMU*: dipole moment, Debye
*ETA*_*i*_: association parameter
*ETA*_*ij*_: solvation parameter
*FF*: fitting function
*g* ^*E*^: excess free energy, J·mol^–1^
*h* ^*E*^: excess molar enthalpy, J·mol^–1^
*m*: number parameters
*MAD*: mean absolute deviation
*n*: counter parameter
*N*: number of experimental data
*OF*: objective function
$p_{i}^{0}$ , $p_{i}^{\mathrm{SAT}}$: vapor pressure for pure substance, kPa
*p*: total pressure, kPa
*p*_C_: critical pressure, kPa
*R*: universal gas constant, J·K^–1^·mol^–1^
*R*_D_: radius of gyration, Å
SD: standard deviation
*T*: temperature, K
*T*_C_: critical temperature, K
*TW*: this work
*V* ^*E*^: excess molar volume, m^3^·mol^–1^
*v*_*i*_^*L*^: molar volume, m^3^·mol^–1^
*x*: liquid-phase mole fraction
*y*: vapor-phase mole fraction
*Z*_*C*_: critical compressibility factor

## Greek symbols

γ: activity coefficient
ϕ: fugacity coefficient
*Δ*_12_, *Δ*_21_: parameters of the correlation of **NRTL** model
α: parameter of mixture in **NRTL** model

## Subscripts, superscripts

cal: calculated
exp: experimental
*i*, *j*: chemical substances
L: liquid phase
m: molar
V: vapor phase
