# Supplementary material for: A Novel Equipment for VLE Studies: Ethanol and Water System at Moderate Pressures and Data Verification with a Direct Point-to-Point Test
Source: ACS Omega. 2026 Aug 5;11(32):48295–312. doi: 10.1021/acsomega.6c04827 (PMC13491997; doi:10.1021/acsomega.6c04827)
Supplement: Supplementary file 1 [file ao6c04827_si_001.pdf]

SUPPORTING INFORMATION

A NOVEL EQUIPMENT FOR VLE STUDIES: ETHANOL AND WATER SYSTEM AT MODERATE PRESSURES AND DATA VERIFICATION WITH A DIRECT POINT-TO-POINT TEST.

Autores:
Pedro Susial Badajoz*, Isabel Garcia Montesdeoca, Dunia E. Santiago

AUTHOR INFORMATION
Authors
Isabel Garcia Montesdeoca
Universidad de Las Palmas de GranCanaria
Instituto de Estudios Ambientales y Recursos Naturales (iUNAT)
Las Palmas de Gran Canaria, 35017
Canary Islands, Spain
Email: isabel.montesdeoca@ulpgc.es; Tfno: 928451876

Dunia E. Santiago
Universidad de Las Palmas de GranCanaria
Instituto de Estudios Ambientales y Recursos Naturales (iUNAT)
Las Palmas de Gran Canaria, 35017
Canary Islands, Spain
Email: dunia.santiago@ulpgc.es;Tfno: 928457301

Corresponding Author
Pedro Susial Badajoz
Universidad de Las Palmas de GranCanaria
Instituto de Estudios Ambientales y Recursos Naturales (iUNAT)
Las Palmas de Gran Canaria, 35017
Canary Islands, Spain
Email: pedro.susial@ulpgc.es; Tfno: 928454437

Table S1. Results of consistency tests on isobaric VLE binary systems of ethanol (1) + water (2) using Antoine constants of this work (TW)

	VLE data			Fredenslund et al.[24]test					PTDGE–h^E_{EXP}					PTDGE				
	References	p/kPa	N data	%p>75	MAD p/kPa	%y_1>75	MAD y_1	TEST	%p>75	MAD p/kPa	%y_1>75	MAD y_1	TEST	%p>75	MAD p/kPa	%y_1>75	MAD y_1	TEST
1	TW	101	43	100.0	0.27<1.51	95.3	0.0045<0.01	P	90.7	1.00<1.51	83.7	0.0044<0.01	P	83.7	1.00<1.51	81.4	0.0055<0.01	P
2		300	20	100.0	0.27<4.50	100.0	0.0042<0.01	P	80.0	3.64<4.50	100.0	0.0051<0.01	P	90.0	3.15<4.50	100.0	0.0042<0.01	P
3		500	22	95.5	2.72<7.50	54.5	0.0095<0.01	S	59.1	6.49<7.50	81.8	0.0074<0.01	S	63.6	5.67<7.50	90.9	0.0064<0.01	S
4		700	22	100.0	3.39<10.50	63.6	0.0111>0.01	N	100.0	5.47<10.50	63.6	0.0089.01	S	100.0	6.17<10.50	77.3	0.0080<0.01	P
5	Susial et al. in *J. Chem. Eng. Data* **2021**, 66, 928−946	100	26	100.0	0.61<1.50	80.8	0.0063<0.01	P	88.5	0.87<1.50	100.0	0.0040<0.01	P	80.8	1.06<1.50	96.2	0.0039<0.01	P
6	Bloom et al. in Gmehling, J. et al. vol 1, part 1, page 153	101.3	18	94.4	0.55<1.52	83.3	0.0094<0.01	P	44.4	2.19<1.52	66.7	0.0076.01	N	44.4	1.95>1.52	72.2	0.0079<0.01	N
7	Carey&Lewis in Gmehling, J. et al. vol 1, part 1, page 154	101.3	15	100.0	0.42<1.52	86.7	0.0074<0.01	P	80.0	1.13<1.52	100.0	0.0025<0.01	P	93.3	0.84<1.52	100.0	0.0037<0.01	P
8	Johnson&Furter in Gmehling, J. et al. vol 1, part 1, page 162	100.7	13	100.0	0.76<1.51	92.3	0.0070<0.01	P	46.2	2.29>1.51	46.2	0.0103>0.01	N	53.8	2.02>1.51	61.5	0.0084<0.01	N
9	Jones et al. in Gmehling, J. et al. vol 1, part 1, page 165	101.3	11	100.0	0.42<1.52	81.8	0.0078<0.01	P	45.5	1.90>1.52	90.9	0.0067<0.01	N	54.5	1.59>1.52	72.7	0.0059<0.01	N
10	Kirschbaum&Gerstner in Gmehling, J. et al. vol 1, part 1, page 167	13.3	11	100.0	0.02<0.20	54.5	0.0119>0.01	N	72.7	0.16<0.20	72.7	0.0123>0.01	N	72.7	0.17<0.20	72.7	0.0131>0.01	N
11	Kirschbaum&Gerstner in Gmehling, J. et al. vol 1, part 1A, page 130	33.3	28	96.4	0.16<0.50	42.9	0.0129>0.01	N	7.1	1.13>0.50	92.9	0.0050<0.01	N	28.6	1.00>0.50	82.1	0.0065<0.01	N
12	Kirschbaum et al. Gmehling, J. et al. vol 1, part 1, page 169	66.7	11	100.0	0.11<1.00	90.9	0.0066<0.01	P	100.0	0.61<1.00	90.9	0.0046<0.01	P	100.0	0.66<1.00	90.9	0.0062<0.01	P
13	Kirschbaum&Gerstner in Gmehling, J. et al. vol 1, part 1A, page 131	66.7	21	100.0	0.16<1.00	85.7	0.0060<0.01	P	100.0	0.59<1.00	95.2	0.0049<0.01	P	100.0	0.55<1.00	90.5	0.0056<0.01	P
14	Kirschbaum&Gerstner in Gmehling, J. et al. vol 1, part 1, page 170	98.7	11	100.0	0.73<1.48	81.8	0.0067<0.01	P	72.7	1.23<1.48	100.0	0.0017<0.01	S	63.6	1.39<1.48	100.0	0.0027<0.01	S
15	Kirschbaum&Gerstner in Gmehling, J. et al. vol 1, part 1A, page 132	98.7	18	100.0	0.43<1.48	77.8	0.0062<0.01	P	83.3	1.02<1.48	100.0	0.0041<0.01	P	83.3	1.02<1.48	100.0	0.0044<0.01	P
16	Kojima et al. in Gmehling, J. et al. vol 1, part 1, page 171	101.3	19	100.0	0.41<1.52	84.2	0.0055<0.01	P	94.7	0.78<1.52	89.5	0.0045<0.01	P	89.5	0.90<1.52	89.5	0.0041<0.01	P
17	Otsuki&Williams in *Chem. Eng. Prog. Symp. Ser.* **1953**, 49, 55–67	101.3	51	96.1	0.50<1.52	92.2	0.0042<0.01	P	88.2	0.78<1.52	94.1	0.0038<0.01	P	86.3	0.85<1.52	98.0	0.0036<0.01	P
18	Otsuki&Williams in *Chem. Eng. Prog. Symp. Ser.* **1953**, 49, 55–67	344.7	21	95.2	1.81<5.17	52.4	0.0109>0.01	N	38.1	6.42>5.17	81.0	0.0064<0.01	N	19.0	7.45>5.17	81.0	0.0056<0.01	N
19	Otsuki&Williams in *Chem. Eng. Prog. Symp. Ser.* **1953**, 49, 55–67	689.5	19	100.0	2.40<10.33	52.6	0.0108>0.01	N	78.9	7.77<10.33	73.7	0.0076<0.01	S	63.2	9.57<10.33	73.7	0.0078<0.01	S
20	Paul, R.N. in Gmehling, J. et al. vol 1, part 1, page 180	101.3	13	84.6	0.92<1.52	46.2	0.0140>0.01	N	84.6	1.60>1.52	100.0	0.0039<0.01	S	84.6	1.30<1.52	92.3	0.0041<0.01	P
21	Rieder&Thompson in Gmehling, J. et al. vol 1, part 1, page 181–182	101.3	34	97.1	0.58<1.52	82.4	0.0050<0.01	P	91.2	0.89<1.52	82.4	0.0067<0.01	P	94.1	0.71<1.52	82.4	0.0056<0.01	P
22	Rius et al. in Gmehling, J. et al. vol 1, part 1, page 183	94.1	12	100.0	0.72<1.41	83.3	0.0096<0.01	P	41.7	1.92>1.41	91.7	0.0043<0.01	N	66.7	1.50>1.41	91.7	0.0049<0.01	N
23	Stabnikov et al. in Gmehling, J. et al. vol 1, part 1, page 184–186	101.3	126	84.1	0.66<1.52	97.6	0.0039<0.01	P	87.3	0.82<1.52	100.0	0.0026<0.01	P	83.3	0.81<1.52	98.4	0.0027<0.01	P
24	Stabnikov et al. in Gmehling, J. et al. vol 1, part 1, page 187	50.7	13	84.6	0.37<0.76	76.9	0.0067<0.01	P	76.9	0.51<0.76	100.0	0.0047<0.01	P	84.6	0.45<0.76	84.6	0.0054<0.01	P
25	Stabnikov et al. in Gmehling, J. et al. vol 1, part 1, page 188	101.3	14	92.9	0.86<1.52	85.7	0.0060<0.01	P	92.9	1.17<1.52	85.7	0.0045<0.01	P	85.7	1.25<1.52	100.0	0.0041<0.01	P
26	Van Zandijcke&Verhoeye in Gmehling, J. et al. vol 1, part 1, page 193	101.3	13	100.0	0.46<1.52	69.2	0.0099<0.01	S	100.0	0.45<1.52	76.9	0.0084<0.01	P	100.0	0.51<1.52	76.9	0.0071<0.01	P
27	Kojima et al. in Gmehling, J. et al. vol 1, part 1A, page 133	101.3	19	100.0	0.09<1.52	100.0	0.0039<0.01	P	100.0	0.71<1.52	100.0	0.0041<0.01	P	100.0	0.70<1.52	89.5	0.0053<0.01	P
28	Macarron, A. in Gmehling, J. et al. vol 1, part 1A, page 136–137	94.0	33	97.0	0.64<1.41	72.7	0.0092<0.01	S	45.5	1.83>1.41	90.9	0.0042<0.01	N	63.6	1.45>1.41	87.9	0.0052<0.01	N
29	Hughes&Maloney in Gmehling, J. et al. vol 1, part 1B, page 91	101.3	19	94.7	0.47<1.52	78.9	0.0064<0.01	P	63.2	1.83>1.52	89.5	0.0056<0.01	N	84.2	1.47<1.52	94.7	0.0050<0.01	P
30	Kauer&Bittrich in Gmehling, J. et al. vol 1, part 1B, page 92	101.3	8	100.0	0.37<1.52	50.0	0.0147>0.01	N	50.0	2.78>1.52	87.5	0.0075<0.01	N	50.0	2.24>1.52	75.0	0.0091<0.01	N
31	Protsyuk&Devyatko in Gmehling, J. et al. vol 1, part 1B, page 109	101.3	14	92.9	0.73<1.52	42.9	0.0207>0.01	N	35.7	3.43>1.52	92.9	0.0048<0.01	N	35.7	3.11>1.52	92.9	0.0037<0.01	N
32	Rao et al. Gmehling, J. et al. vol 1, part 1B, page 110	101.3	9	100.0	0.53<1.52	77.8	0.0061<0.01	P	88.9	0.85<1.52	100.0	0.0047<0.01	P	88.9	0.62<1.52	88.9	0.0068<0.01	P
33	Schmitt, D. in Gmehling, J. et al. vol 1, part 1B, page 111	100.7	19	100.0	0.64<1.51	94.7	0.0057<0.01	P	68.4	1.57>1.51	94.7	0.0032<0.01	N	68.4	1.55>1.51	94.7	0.0031<0.01	N
34	Arce et al. in Gmehling, J. et al. vol 1, part 1C, page 176	101.3	21	100.0	0.32<1.52	71.4	0.0086<0.01	S	42.9	2.10>1.52	95.2	0.0051<0.01	N	42.9	1.88>1.52	90.5	0.0064<0.01	N
35	Chen et al. in Gmehling, J. et al. vol 1, part 1C, page 179	101.3	10	100.0	0.73<1.52	60.0	0.0145>0.01	N	80.0	1.46<1.52	90.0	0.0068<0.01	P	90.0	1.29<1.52	90.0	0.0066<0.01	P
36	Chou et al. in Gmehling, J. et al. vol 1, part 1C, page 180	101.3	15	100.0	0.37<1.52	93.3	0.0046<0.01	P	93.3	0.56<1.52	86.7	0.0040<0.01	P	93.3	0.65<1.52	86.7	0.0053<0.01	P
37	Cui et al. in Gmehling, J. et al. vol 1, part 1C, page 183	101.3	9	100.0	0.73<1.52	77.8	0.0085<0.01	P	55.6	1.47<1.52	100.0	0.0054<0.01	S	77.8	1.51<1.52	88.9	0.0054<0.01	P
38	Iwakabe&Kosugue in Gmehling, J. et al. vol 1, part 1C, page 196	101.3	28	100.0	0.33<1.52	96.4	0.0031<0.01	P	100.0	0.39<1.52	85.7	0.0035<0.01	P	100.0	0.48<1.52	89.3	0.0049<0.01	P
39	Kurihara et al. in Gmehling, J. et al. vol 1, part 1C, page 204	101.3	18	100.0	0.12<1.52	72.2	0.0081<0.01	S	77.8	0.97<1.52	72.2	0.0079<0.01	S	72.2	1.03<1.52	72.2	0.0093<0.01	S
40	Kraehenbuehl, M.A. in Gmehling, J. et al. vol 1, part 1C, page 211	20.0	20	100.0	0.08<0.30	40.0	0.0126>0.01	N	20.0	0.63>0.30	75.0	0.0077<0.01	N	15.0	0.61>0.30	70.0	0.0082<0.01	N
41	Kraehenbuehl, M.A. in Gmehling, J. et al. vol 1, part 1C, page 212	40.0	17	100.0	0.14<0.60	64.7	0.0096<0.01	S	58.8	0.63>0.60	58.8	0.0092<0.01	N	52.9	0.60=0.60	64.7	0.0104>0.01	N
42	Kraehenbuehl, M.A. in Gmehling, J. et al. vol 1, part 1C, page 213	50.7	26	100.0	0.20<0.76	30.8	0.0148>0.01	N	46.2	0.83>0.76	50.0	0.0105>0.01	N	53.8	0.80>0.76	42.3	0.0128>0.01	N
43	Kraehenbuehl, M.A. in Gmehling, J. et al. vol 1, part 1C, page 215	80.0	16	87.5	0.37<1.20	56.2	0.0115>0.01	N	81.2	0.71<1.20	68.8	0.0082<0.01	S	75.0	0.88<1.20	62.5	0.0092<0.01	S
44	Kraehenbuehl, M.A. in Gmehling, J. et al. vol 1, part 1C, page 216	101.3	13	100.0	0.37<1.52	53.8	0.0113>0.01	N	69.2	1.14<1.52	69.2	0.0120>0.01	S	76.9	1.17<1.52	46.2	0.0138>0.01	N
45	Kraehenbuehl, M.A. in Gmehling, J. et al. vol 1, part 1C, page 217	120.0	13	100.0	0.55<1.80	61.5	0.0116>0.01	N	23.1	7.76>1.80	23.1	0.0268>0.01	N	23.1	7.91>1.80	23.1	0.0274>0.01	N
46	Zemp&Francesconi in Gmehling, J. et al. vol 1, part 1C, page 252	101.3	24	91.7	0.58<1.52	58.3	0.0112>0.01	N	29.2	2.90>1.52	79.2	0.0071<0.01	N	25.0	2.87>1.52	79.2	0.0071<0.01	N
47	Alvarez et al. in *Phys. Chem. Liq.* **2011**, 49, 52–71	101.3	36	100.0	0.23<1.52	19.4	0.0155>0.01	N	0.0	4.21>1.52	80.6	0.0066<0.01	N	0.0	4.01>1.52	72.2	0.0072<0.01	N
48	Voutsas et al. in *Fluid Phase Equilib.* **2011**, 308, 135–141	13.15	15	100.0	0.02<0.20	86.7	0.0060<0.01	P	40.0	0.22>0.20	93.3	0.0037<0.01	N	40.0	0.21>0.20	93.3	0.0044<0.01	N
49	Voutsas et al. in *Fluid Phase Equilib.* **2011**, 308, 135–141	19.71	16	100.0	0.08<0.30	68.8	0.0112>0.01	N	31.2	0.35>0.30	68.8	0.0074<0.01	N	31.2	0.33>0.30	68.8	0.0084<0.01	N
50	Voutsas et al. in *Fluid Phase Equilib.* **2011**, 308, 135–141	32.86	14	85.7	0.30<0.49	78.6	0.0071<0.01	P	92.9	0.41<0.49	85.7	0.0058<0.01	P	92.9	0.36<0.49	85.7	0.0060<0.01	P
51	Othmer et al in Gmehling, J. et al. vol 1, part 1A, page 139	287.4	13	38.5	8.69>4.31	30.8	0.0178>0.01	N	15.4	14.69>4.31	46.2	0.0108>0.01	N	15.4	15.04>4.31	46.2	0.0109>0.01	N
52	Othmer et al in Gmehling, J. et al. vol 1, part 1, page 176	515.0	11	90.9	4.47<7.72	18.2	0.0322>0.01	N	72.7	12.94>7.72	18.2	0.0322>0.01	N	72.7	13.06>7.72	18.2	0.0314>0.01	N
53	Othmer et al in Gmehling, J. et al. vol 1, part 1, page 175	721.8	11	72.7	14.73>10.83	18.2	0.0353>0.01	N	0.0	57.95>10.83	72.7	0.0094<0.01	N	0.0	59.50>10.83	81.8	0.0079<0.01	N

Table S2. Results of consistency tests on isobaric VLE binary systems of ethanol (1) + water (2) using Antoine constants of D$D[22]

	VLE data			Fredenslund et al.[24] test					PTDGE–h^E_{EXP}					PTDGE				
	References	p/kPa	N data	%p>75	*MAD* p/kPa	%y_1>75	*MAD* y_1	TEST	%p>75	*MAD* p/kPa	%y_1>75	*MAD* y_1	TEST	%p>75	*MAD* p/kPa	%y_1>75	*MAD* y_1	TEST
1	TW	101	43	100.0	0.30<1.51	100.0	0.0030<0.01	P	86.0	0.79<1.51	90.7	0.0038<0.01	P	81.4	0.95<1.51	86.0	0.0047<0.01	P
2		300	20	100.0	0.24<4.50	95.0	0.0056<0.01	P	75.0	4.12<4.50	80.0	0.0074<0.01	P	85.0	3.63<4.50	100.0	0.0061<0.01	P
3		500	22	95.5	2.79<7.50	36.4	0.0104>0.01	N	40.9	8.43>7.50	50.0	0.0098<0.01	N	45.5	7.59>7.50	68.2	0.0087<0.01	N
4		700	22	100.0	3.13<10.50	54.5	0.0124>0.01	N	100.0	3.23<10.50	54.5	0.0113>0.01	N	100.0	3.57<10.50	68.2	0.0103>0.01	N
5	Susial et al. in *J. Chem. Eng. Data* **2021**, 66, 928−946	100	26	100.0	0.56<1.50	53.8	0.0100=0.01	S	65.4	1.35<1.50	96.2	0.0046<0.01	S	65.4	1.63>1.50	92.3	0.0043<0.01	N
6	Bloom et al. in Gmehling, J. et al. vol 1, part 1, page 153	101.3	18	94.4	0.59<1.52	72.2	0.0093<0.01	S	66.7	1.43<1.52	72.2	0.0077<0.01	S	61.1	1.28<1.52	66.7	0.0078<0.01	S
7	Carey&Lewis in Gmehling, J. et al. vol 1, part 1, page 154	101.3	15	100.0	0.37<1.52	66.7	0.0087<0.01	S	80.0	1.05<1.52	100.0	0.0024<0.01	P	86.7	1.08<1.52	100.0	0.0032<0.01	P
8	Johnson&Furter in Gmehling, J. et al. vol 1, part 1, page 162	100.7	13	84.6	0.85<1.51	84.6	0.0066<0.01	P	69.2	1.24<1.51	61.5	0.0104>0.01	N	69.2	1.16<1.51	76.9	0.0086<0.01	S
9	Jones et al. in Gmehling, J. et al. vol 1, part 1, page 165	101.3	11	100.0	0.46<1.52	81.8	0.0081<0.01	P	90.9	1.16<1.52	100.0	0.0064<0.01	P	90.9	0.93<1.52	81.8	0.0056<0.01	P
10	Kirschbaum&Gerstner in Gmehling, J. et al. vol 1, part 1, page 167	13.3	11	90.9	0.11<0.20	54.5	0.0146>0.01	N	0.0	0.79>0.20	72.7	0.0112>0.01	N	0.0	0.82<0.20	72.7	0.0120>0.01	N
11	Kirschbaum&Gerstner in Gmehling, J. et al. vol 1, part 1A, page 130	33.3	28	100.0	0.10<0.50	89.3	0.0062<0.01	P	75.0	0.36<0.50	89.3	0.0051<0.01	P	53.6	0.48<0.50	78.6	0.0065<0.01	S
12	Kirschbaum et al. Gmehling, J. et al. vol 1, part 1, page 169	66.7	11	100.0	0.23<1.00	90.9	0.0053<0.01	P	81.8	0.89<1.00	90.9	0.0039<0.01	P	81.8	1.05>1.00	90.9	0.0046<0.01	N
13	Kirschbaum&Gerstner in Gmehling, J. et al. vol 1, part 1A, page 131	66.7	21	95.2	0.21<1.00	61.9	0.0095<0.01	S	71.4	1.03>1.00	95.2	0.0050<0.01	N	42.9	1.28>1.00	90.5	0.0056<0.01	N
14	Kirschbaum&Gerstner in Gmehling, J. et al. vol 1, part 1, page 170	98.7	11	90.9	0.90<1.48	36.4	0.0134>0.01	N	36.4	2.56>1.48	100.0	0.0025<0.01	N	18.2	2.79>1.48	100.0	0.0024<0.01	N
15	Kirschbaum&Gerstner in Gmehling, J. et al. vol 1, part 1A, page 132	98.7	18	100.0	0.35<1.48	66.7	0.0101>0.01	N	50.0	1.82>1.48	100.0	0.0039<0.01	N	27.8	2.03>1.48	100.0	0.0040<0.01	N
16	Kojima et al. in Gmehling, J. et al. vol 1, part 1, page 171	101.3	19	100.0	0.30<1.52	78.9	0.0086<0.01	P	89.5	0.80<1.52	84.2	0.0052<0.01	P	73.7	1.00<1.52	89.5	0.0048<0.01	S
17	Otsuki&Williams in *Chem. Eng. Prog. Symp. Ser.* **1953**, 49, 55–67	101.3	51	96.1	0.45<1.52	76.5	0.0079<0.01	P	84.3	1.03<1.52	92.2	0.0042<0.01	P	66.7	1.33<1.52	96.1	0.0037<0.01	S
18	Otsuki&Williams in *Chem. Eng. Prog. Symp. Ser.* **1953**, 49, 55–67	344.7	21	95.2	2.73<5.17	57.1	0.0122>0.01	N	42.9	6.21>5.17	71.4	0.0071<0.01	N	19.0	7.32>5.17	81.0	0.0058<0.01	N
19	Otsuki&Williams in *Chem. Eng. Prog. Symp. Ser.* **1953**, 49, 55–67	689.5	19	100.0	2.41<10.33	36.8	0.0117>0.01	N	89.5	5.21<10.33	63.2	0.0096<0.01	S	73.7	6.93<10.33	68.4	0.0096<0.01	S
20	Paul, R.N. in Gmehling, J. et al. vol 1, part 1, page 180	101.3	13	76.9	0.97<1.52	23.1	0.0151>0.01	N	61.5	1.72>1.52	92.3	0.0047<0.01	N	61.5	1.75>1.52	92.3	0.0047<0.01	N
21	Rieder&Thompson in Gmehling, J. et al. vol 1, part 1, page 181–182	101.3	34	94.1	0.58<1.52	73.5	0.0094<0.01	S	79.4	0.83<1.52	79.4	0.0066<0.01	P	70.6	1.07<1.52	82.4	0.0054<0.01	S
22	Rius et al. in Gmehling, J. et al. vol 1, part 1, page 183	94.1	12	100.0	0.62<1.41	91.7	0.0046<0.01	P	83.3	0.70<1.41	100.0	0.0039<0.01	P	100.0	0.74<1.41	91.7	0.0047<0.01	P
23	Stabnikov et al. in Gmehling, J. et al. vol 1, part 1, page 184–186	101.3	126	87.3	0.61<1.52	79.4	0.0056<0.01	P	84.1	1.05<1.52	100.0	0.0033<0.01	P	77.8	1.24<1.52	99.2	0.0029<0.01	P
24	Stabnikov et al. in Gmehling, J. et al. vol 1, part 1, page 187	50.7	13	100.0	0.37<0.76	61.5	0.0101>0.01	N	30.8	1.17>0.76	100.0	0.0041<0.01	N	23.1	1.24>0.76	92.3	0.0046<0.01	N
25	Stabnikov et al. in Gmehling, J. et al. vol 1, part 1, page 188	101.3	14	92.9	0.88<1.52	78.6	0.0069<0.01	P	71.4	1.18<1.52	85.7	0.0048<0.01	S	71.4	1.42<1.52	92.9	0.0041<0.01	S
26	Van Zandijcke&Verhoeye in Gmehling, J. et al. vol 1, part 1, page 193	101.3	13	100.0	0.38<1.52	30.8	0.0151>0.01	N	69.2	1.31<1.52	53.8	0.0100=0.01	S	69.2	1.52=1.52	69.2	0.0085<0.01	S
27	Kojima et al. in Gmehling, J. et al. vol 1, part 1A, page 133	101.3	19	100.0	0.16<1.52	100.0	0.0024<0.01	P	100.0	0.67<1.52	100.0	0.0032<0.01	P	89.5	0.82<1.52	94.7	0.0043<0.01	P
28	Macarron, A. in Gmehling, J. et al. vol 1, part 1A, page 136–137	94.0	33	97.0	0.60<1.41	90.9	0.0045<0.01	P	72.7	1.05<1.41	93.9	0.0039<0.01	S	72.7	0.99<1.41	93.9	0.0048<0.01	S
29	Hughes&Maloney in Gmehling, J. et al. vol 1, part 1B, page 91	101.3	19	100.0	0.39<1.52	78.9	0.0081<0.01	P	89.5	1.03<1.52	94.7	0.0056<0.01	P	89.5	1.17<1.52	89.5	0.0049<0.01	P
30	Kauer&Bittrich in Gmehling, J. et al. vol 1, part 1B, page 92	101.3	8	100.0	0.34<1.52	62.5	0.0084<0.01	S	37.5	2.04>1.52	87.5	0.0075<0.01	N	62.5	1.83>1.52	75.0	0.0088<0.01	N
31	Protsyuk&Devyatko in Gmehling, J. et al. vol 1, part 1B, page 109	101.3	14	85.7	0.73<1.52	28.6	0.0208>0.01	N	50.0	2.86>1.52	85.7	0.0061<0.01	N	50.0	2.58>1.52	92.9	0.0046<0.01	N
32	Rao et al. Gmehling, J. et al. vol 1, part 1B, page 110	101.3	9	100.0	0.56<1.52	88.9	0.0079<0.01	P	77.8	1.62>1.52	100.0	0.0031<0.01	N	88.9	1.66>1.52	100.0	0.0052<0.01	N
33	Schmitt, D. in Gmehling, J. et al. vol 1, part 1B, page 111	100.7	19	100.0	0.38<1.51	94.7	0.0036<0.01	P	89.5	0.68<1.51	89.5	0.0035<0.01	P	89.5	0.70<1.51	89.5	0.0032<0.01	P
34	Arce et al. in Gmehling, J. et al. vol 1, part 1C, page 176	101.3	21	100.0	0.25<1.52	95.2	0.0061<0.01	P	66.7	0.96<1.52	95.2	0.0038<0.01	S	81.0	0.77<1.52	95.2	0.0048<0.01	P
35	Chen et al. in Gmehling, J. et al. vol 1, part 1C, page 179	101.3	10	100.0	0.82<1.52	60.0	0.0171>0.01	N	60.0	1.84>1.52	90.0	0.0057<0.01	N	50.0	1.78>1.52	90.0	0.0055<0.01	N
36	Chou et al. in Gmehling, J. et al. vol 1, part 1C, page 180	101.3	15	100.0	0.36<1.52	86.7	0.0061<0.01	P	73.3	1.27<1.52	86.7	0.0038<0.01	S	73.3	1.52=1.52	86.7	0.0046<0.01	S
37	Cui et al. in Gmehling, J. et al. vol 1, part 1C, page 183	101.3	9	100.0	0.77<1.52	77.8	0.0097<0.01	P	66.7	1.56>1.52	100.0	0.0053<0.01	N	66.7	1.86>1.52	100.0	0.0052<0.01	N
38	Iwakabe&Kosugue in Gmehling, J. et al. vol 1, part 1C, page 196	101.3	28	100.0	0.27<1.52	85.7	0.0074<0.01	P	89.3	1.19<1.52	89.3	0.0039<0.01	P	67.9	1.54>1.52	85.7	0.0047<0.01	N
39	Kurihara et al. in Gmehling, J. et al. vol 1, part 1C, page 204	101.3	18	100.0	0.19<1.52	88.9	0.0054<0.01	P	88.9	0.82<1.52	88.9	0.0062<0.01	P	88.9	0.89<1.52	72.2	0.0075<0.01	S
40	Kraehenbuehl, M.A. in Gmehling, J. et al. vol 1, part 1C, page 211	20.0	20	95.0	0.12<0.30	65.0	0.0096<0.01	S	50.0	0.40>0.30	85.0	0.0078<0.01	N	50.0	0.45>0.30	70.0	0.0080<0.01	N
41	Kraehenbuehl, M.A. in Gmehling, J. et al. vol 1, part 1C, page 212	40.0	17	82.4	0.40<0.60	47.1	0.0105>0.01	N	41.2	0.86>0.60	58.8	0.0089<0.01	N	35.3	0.97>0.60	64.7	0.0099<0.01	N
42	Kraehenbuehl, M.A. in Gmehling, J. et al. vol 1, part 1C, page 213	50.7	26	100.0	0.21<0.76	57.7	0.0102>0.01	N	65.4	0.68<0.76	57.7	0.0100=0.01	S	57.7	0.85>0.76	42.3	0.0122>0.01	N
43	Kraehenbuehl, M.A. in Gmehling, J. et al. vol 1, part 1C, page 215	80.0	16	87.5	0.54<1.20	50.0	0.0128>0.01	N	6.2	2.07>1.20	68.8	0.0078<0.01	N	6.2	2.24>1.20	62.5	0.0086<0.01	N
44	Kraehenbuehl, M.A. in Gmehling, J. et al. vol 1, part 1C, page 216	101.3	13	100.0	0.41<1.52	84.6	0.0077<0.01	P	84.6	0.93<1.52	69.2	0.0106>0.01	N	76.9	1.06<1.52	61.5	0.0123>0.01	N
45	Kraehenbuehl, M.A. in Gmehling, J. et al. vol 1, part 1C, page 217	120.0	13	100.0	0.82<1.80	69.2	0.0098<0.01	S	23.1	7.39>1.80	23.1	0.0269>0.01	N	23.1	7.66>1.80	30.8	0.0274>0.01	N
46	Zemp&Francesconi in Gmehling, J. et al. vol 1, part 1C, page 252	101.3	24	95.8	0.39<1.52	70.8	0.0039<0.01	S	41.7	2.12>1.52	83.3	0.0075<0.01	N	45.8	2.09>1.52	79.2	0.0073<0.01	N
47	Alvarez et al. in *Phys. Chem. Liq.* **2011**, 49, 52–71	101.3	36	100.0	0.19<1.52	75.0	0.0097<0.01	P	27.8	2.95>1.52	80.6	0.0064<0.01	N	33.3	2.74>1.52	75.0	0.0069<0.01	N
48	Voutsas et al. in *Fluid Phase Equilib.* **2011**, 308, 135–141	13.15	15	93.3	0.07<0.20	46.7	0.0124>0.01	N	0.0	0.63>0.20	100.0	0.0031<0.01	N	0.0	0.64>0.20	93.3	0.0038<0.01	N
49	Voutsas et al. in *Fluid Phase Equilib.* **2011**, 308, 135–141	19.71	16	100.0	0.08<0.30	62.5	0.0091<0.01	S	6.2	0.66>0.30	81.2	0.0065<0.01	N	0.0	0.68>0.30	68.8	0.0075<0.01	N
50	Voutsas et al. in *Fluid Phase Equilib.* **2011**, 308, 135–141	32.86	14	100.0	0.22<0.49	35.7	0.0125>0.01	N	7.1	0.92>0.49	85.7	0.0060<0.01	N	7.1	0.98>0.49	85.7	0.0061<0.01	N
51	Othmer et al in Gmehling, J. et al. vol 1, part 1A, page 139	287.4	13	38.5	8.78>4.31	30.8	0.0183>0.01	N	15.4	14.45>4.31	69.2	0.0100=0.01	N	15.4	14.98>4.31	69.2	0.0101>0.01	N
52	Othmer et al in Gmehling, J. et al. vol 1, part 1, page 176	515.0	11	36.4	10.79>7.72	18.2	0.0266>0.01	N	72.7	12.47>7.72	18.2	0.0336>0.01	N	72.7	11.93>7.72	18.2	0.0327>0.01	N
53	Othmer et al in Gmehling, J. et al. vol 1, part 1, page 175	721.8	11	36.4	14.95>10.83	9.1	0.0370>0.01	N	0.0	53.57>10.83	63.6	0.0121>0.01	N	0.0	55.70>10.83	63.6	0.0105>0.01	N

Table S3. Results of consistency tests on isobaric VLE binary systems of ethanol (1) + water (2) using Antoine constants of VDI[23]

	VLE data			Fredenslund et al.[24] test					PTDGE–h^E_{EXP}					PTDGE				
	References	p/kPa	N data	%p>75	*MAD p*/kPa	%y_1>75	*MAD* y_1	TEST	%p>75	*MAD p*/kPa	%y_1>75	*MAD* y_1	TEST	%p>75	*MAD p*/kPa	%y_1>75	*MAD* y_1	TEST
1	TW	101	43	100.0	0.28<1.51	97.7	0.0044<0.01	P	88.4	0.84<1.51	90.7	0.0037<0.01	P	83.7	0.97<1.51	88.4	0.0045<0.01	P
2		300	20	100.0	0.29<4.50	100.0	0.0053<0.01	P	90.0	2.87<4.50	85.0	0.0061<0.01	P	100.0	2.46<4.50	100.0	0.0050<0.01	P
3		500	22	100.0	2.63<7.50	45.5	0.0098<0.01	S	68.2	5.99<7.50	77.3	0.0079<0.01	S	81.8	5.19<7.50	86.4	0.0069<0.01	P
4		700	22	100.0	3.33<10.50	54.5	0.0116>0.01	N	100.0	5.59<10.50	63.6	0.0093<0.01	S	95.5	6.64<10.50	72.7	0.0083<0.01	S
5	Susial et al. in *J. Chem. Eng. Data* **2021**, 66, 928−946	100	26	100.0	0.63<1.50	53.8	0.00100=0.01	S	69.2	1.29<1.50	92.3	0.0054<0.01	S	65.4	1.56>1.50	92.3	0.0048<0.01	N
6	Bloom et al. in Gmehling, J. et al. vol 1, part 1, page 153	101.3	18	94.4	0.55<1.52	72.2	0.0090<0.01	S	66.7	1.67>1.52	72.2	0.0081<0.01	N	61.1	1.51<1.52	72.2	0.0080<0.01	S
7	Carey&Lewis in Gmehling, J. et al. vol 1, part 1, page 154	101.3	15	100.0	0.41<1.52	80.0	0.0082<0.01	P	93.3	0.81<1.52	100.0	0.0029<0.01	P	93.3	0.75<1.52	100.0	0.0032<0.01	P
8	Johnson&Furter in Gmehling, J. et al. vol 1, part 1, page 162	100.7	13	84.6	1.04<1.51	76.9	0.0086<0.01	P	53.8	1.91>1.51	53.8	0.0106>0.01	N	61.5	1.80>1.51	76.9	0.0088<0.01	N
9	Jones et al. in Gmehling, J. et al. vol 1, part 1, page 165	101.3	11	100.0	0.42<1.52	90.9	0.0079<0.01	P	90.9	1.04<1.52	100.0	0.0068<0.01	P	90.9	0.78<1.52	90.9	0.0058<0.01	P
10	Kirschbaum&Gerstner in Gmehling, J. et al. vol 1, part 1, page 167	13.3	11	100.0	0.02<0.20	90.9	0.0087<0.01	P	63.6	0.22>0.20	81.8	0.0067<0.01	N	63.6	0.24>0.20	72.7	0.0068<0.01	N
11	Kirschbaum&Gerstner in Gmehling, J. et al. vol 1, part 1A, page 130	33.3	28	100.0	0.12<0.50	85.7	0.0054<0.01	P	78.6	0.39>0.50	85.7	0.0058<0.01	P	67.9	0.48<0.50	75.0	0.0069<0.01	S
12	Kirschbaum et al. Gmehling, J. et al. vol 1, part 1, page 169	66.7	11	100.0	0.13<1.00	90.9	0.0045<0.01	P	100.0	0.46<1.00	100.0	0.0041<0.01	P	81.8	0.58<1.00	90.9	0.0038<0.01	P
13	Kirschbaum&Gerstner in Gmehling, J. et al. vol 1, part 1A, page 131	66.7	21	95.2	0.23<1.00	66.7	0.0090<0.01	S	85.7	0.82<1.00	95.2	0.0055<0.01	P	57.1	1.08>1.00	90.5	0.0058<0.01	N
14	Kirschbaum&Gerstner in Gmehling, J. et al. vol 1, part 1, page 170	98.7	11	100.0	0.70<1.48	54.5	0.0114>0.01	N	54.5	1.72>1.48	100.0	0.0035<0.01	N	54.5	1.95>1.48	100.0	0.0030<0.01	N
15	Kirschbaum&Gerstner in Gmehling, J. et al. vol 1, part 1A, page 132	98.7	18	100.0	0.30<1.48	66.7	0.0098<0.01	S	55.6	1.62>1.48	100.0	0.0038<0.01	N	27.8	1.82>1.48	100.0	0.0038<0.01	N
16	Kojima et al. in Gmehling, J. et al. vol 1, part 1, page 171	101.3	19	100.0	0.25<1.52	78.9	0.0083<0.01	P	89.5	0.92<1.52	78.9	0.0061<0.01	P	84.2	1.09<1.52	84.2	0.0055<0.01	P
17	Otsuki&Williams in *Chem. Eng. Prog. Symp. Ser.* **1953**, 49, 55–67	101.3	51	98.0	0.47<1.52	78.4	0.0074<0.01	P	84.3	0.86<1.52	88.2	0.0048<0.01	P	66.7	1.11<1.52	96.1	0.0039<0.01	S
18	Otsuki&Williams in *Chem. Eng. Prog. Symp. Ser.* **1953**, 49, 55–67	344.7	21	95.2	2.81<5.17	57.1	0.0130>0.01	N	23.8	7.40>5.17	81.0	0.0065<0.01	N	9.5	8.54>5.17	81.0	0.0056<0.01	N
19	Otsuki&Williams in *Chem. Eng. Prog. Symp. Ser.* **1953**, 49, 55–67	689.5	19	100.0	2.31<10.33	36.8	0.0120>0.01	N	73.7	8.88<10.33	73.7	0.0079<0.01	S	63.2	10.71>10.33	73.7	0.0081<0.01	N
20	Paul, R.N. in Gmehling, J. et al. vol 1, part 1, page 180	101.3	13	76.9	0.92<1.52	30.8	0.0142>0.01	N	84.6	1.37<1.52	92.3	0.0054<0.01	P	84.6	1.31<1.52	92.3	0.0054<0.01	P
21	Rieder&Thompson in Gmehling, J. et al. vol 1, part 1, page 181–182	101.3	34	97.1	0.59<1.52	76.5	0.0091<0.01	P	91.2	0.66<1.52	76.5	0.0069<0.01	P	85.3	0.86<1.52	79.4	0.0055<0.01	P
22	Rius et al. in Gmehling, J. et al. vol 1, part 1, page 183	94.1	12	100.0	0.59<1.41	91.7	0.0043<0.01	P	83.3	0.66<1.41	100.0	0.0036<0.01	P	100.0	0.68<1.41	91.7	0.0046<0.01	P
23	Stabnikov et al. in Gmehling, J. et al. vol 1, part 1, page 184–186	101.3	126	87.3	0.58<1.52	80.2	0.0057<0.01	P	85.7	0.70<1.52	100.0	0.0042<0.01	P	80.2	0.89<1.52	100.0	0.0035<0.01	P
24	Stabnikov et al. in Gmehling, J. et al. vol 1, part 1, page 187	50.7	13	92.3	0.30<0.76	76.9	0.0080<0.01	P	92.3	0.44<0.76	100.0	0.0036<0.01	P	84.6	0.46<0.76	100.0	0.0039<0.01	P
25	Stabnikov et al. in Gmehling, J. et al. vol 1, part 1, page 188	101.3	14	92.9	0.81<1.52	78.6	0.0074<0.01	P	78.6	1.03<1.52	85.7	0.0054<0.01	P	71.4	1.23<1.52	92.9	0.0044<0.01	S
26	Van Zandijcke&Verhoeye in Gmehling, J. et al. vol 1, part 1, page 193	101.3	13	100.0	0.42<1.52	46.2	0.0141>0.01	N	100.0	0.63<1.52	53.8	0.0115>0.01	N	92.3	0.80<1.52	61.5	0.0098<0.01	S
27	Kojima et al. in Gmehling, J. et al. vol 1, part 1A, page 133	101.3	19	100.0	0.25<1.52	100.0	0.0024<0.01	P	100.0	0.29<1.52	100.0	0.0028<0.01	P	94.7	0.36<1.52	94.7	0.0035<0.01	P
28	Macarron, A. in Gmehling, J. et al. vol 1, part 1A, page 136–137	94.0	33	97.0	0.60<1.41	87.9	0.0045<0.01	P	78.8	1.00<1.41	93.9	0.0039<0.01	P	75.8	0.92<1.41	93.9	0.0045<0.01	P
29	Hughes&Maloney in Gmehling, J. et al. vol 1, part 1B, page 91	101.3	19	100.0	0.37<1.52	78.9	0.0073<0.01	P	94.7	0.92<1.52	94.7	0.0057<0.01	P	94.7	0.99<1.52	89.5	0.0048<0.01	P
30	Kauer&Bittrich in Gmehling, J. et al. vol 1, part 1B, page 92	101.3	8	100.0	0.61<1.52	75.0	0.0088<0.01	P	50.0	1.80>1.52	87.5	0.0075<0.01	N	62.5	1.55>1.52	87.5	0.0086<0.01	N
31	Protsyuk&Devyatko in Gmehling, J. et al. vol 1, part 1B, page 109	101.3	14	85.7	0.69<1.52	28.6	0.0208>0.01	N	50.0	2.73>1.52	85.7	0.0071<0.01	N	57.1	2.45>1.52	85.7	0.0054<0.01	N
32	Rao et al. Gmehling, J. et al. vol 1, part 1B, page 110	101.3	9	100.0	0.51<1.52	88.9	0.0067<0.01	P	88.9	0.98<1.52	100.0	0.0021<0.01	P	100.0	1.02<1.52	100.0	0.0041<0.01	P
33	Schmitt, D. in Gmehling, J. et al. vol 1, part 1B, page 111	100.7	19	100.0	0.63<1.51	94.7	0.0053<0.01	P	73.7	1.51>1.51	89.5	0.0039<0.01	N	78.9	1.54>1.51	89.5	0.0035<0.01	N
34	Arce et al. in Gmehling, J. et al. vol 1, part 1C, page 176	101.3	21	100.0	0.32<1.52	89.5	0.0059<0.01	P	61.9	1.54>1.52	90.5	0.0032<0.01	N	76.2	1.34<1.52	95.2	0.0037<0.01	P
35	Chen et al. in Gmehling, J. et al. vol 1, part 1C, page 179	101.3	10	100.0	0.86<1.52	20.0	0.0146>0.01	N	90.0	1.53>1.52	90.0	0.0052<0.01	N	90.0	1.44<1.52	90.0	0.0049<0.01	P
36	Chou et al. in Gmehling, J. et al. vol 1, part 1C, page 180	101.3	15	100.0	0.38<1.52	86.7	0.0055<0.01	P	80.0	0.68<1.52	86.7	0.0039<0.01	P	73.3	0.93<1.52	86.7	0.0046<0.01	S
37	Cui et al. in Gmehling, J. et al. vol 1, part 1C, page 183	101.3	9	100.0	0.79<1.52	77.8	0.0099<0.01	P	66.7	1.48<1.52	100.0	0.0052<0.01	S	66.7	1.79>1.52	100.0	0.0051<0.01	N
38	Iwakabe&Kosugue in Gmehling, J. et al. vol 1, part 1C, page 196	101.3	28	100.0	0.37<1.52	85.7	0.0068<0.01	P	89.3	0.80<1.52	89.3	0.0047<0.01	P	75.0	1.13<1.52	85.7	0.0050<0.01	P
39	Kurihara et al. in Gmehling, J. et al. vol 1, part 1C, page 204	101.3	18	100.0	0.30<1.52	83.3	0.0060<0.01	P	94.4	0.77<1.52	100.0	0.0047<0.01	P	88.9	0.83<1.52	83.3	0.0060<0.01	P
40	Kraehenbuehl, M.A. in Gmehling, J. et al. vol 1, part 1C, page 211	20.0	20	100.0	0.09<0.30	80.0	0.0098<0.01	P	30.0	0.45>0.30	60.0	0.0090<0.01	N	25.0	0.47>0.30	65.0	0.0087<0.01	N
41	Kraehenbuehl, M.A. in Gmehling, J. et al. vol 1, part 1C, page 212	40.0	17	100.0	0.20<0.60	76.5	0.0090<0.01	P	64.7	0.58<0.60	70.6	0.0094<0.01	S	52.9	0.66>0.60	70.6	0.0097<0.01	N
42	Kraehenbuehl, M.A. in Gmehling, J. et al. vol 1, part 1C, page 213	50.7	26	100.0	0.25<0.76	57.7	0.0099<0.01	S	73.1	0.50<0.76	76.9	0.0085<0.01	S	65.4	0.63<0.76	57.7	0.0105>0.01	N
43	Kraehenbuehl, M.A. in Gmehling, J. et al. vol 1, part 1C, page 215	80.0	16	93.8	0.39<1.20	56.2	0.0119>0.01	N	75.0	1.27>1.20	87.5	0.0074<0.01	N	68.8	1.45>1.20	75.0	0.0079<0.01	N
44	Kraehenbuehl, M.A. in Gmehling, J. et al. vol 1, part 1C, page 216	101.3	13	100.0	0.38<1.52	76.9	0.0078<0.01	P	100.0	0.91<1.52	69.2	0.0093<0.01	S	84.6	1.00<1.52	61.5	0.0111>0.01	N
45	Kraehenbuehl, M.A. in Gmehling, J. et al. vol 1, part 1C, page 217	120.0	13	92.3	0.85<1.80	69.2	0.0113>0.01	N	23.1	7.78>1.80	23.1	0.0271>0.01	N	23.1	8.01>1.80	23.1	0.0274>0.01	N
46	Zemp&Francesconi in Gmehling, J. et al. vol 1, part 1C, page 252	101.3	24	91.7	0.62<1.52	58.3	0.0099<0.01	S	25.0	2.70>1.52	83.3	0.0079<0.01	N	25.0	2.67>1.52	83.3	0.0074<0.01	N
47	Alvarez et al. in *Phys. Chem. Liq.* **2011**, 49, 52–71	101.3	36	97.2	0.58<1.52	47.2	0.0116>0.01	N	2.8	3.52>1.52	77.8	0.0064<0.01	N	8.3	3.32>1.52	80.6	0.0067<0.01	N
48	Voutsas et al. in *Fluid Phase Equilib.* **2011**, 308, 135–141	13.15	15	100.0	0.05<0.20	80.0	0.0086<0.01	P	33.3	0.24>0.20	86.7	0.0049<0.01	N	33.3	0.23>0.20	93.3	0.0041<0.01	N
49	Voutsas et al. in *Fluid Phase Equilib.* **2011**, 308, 135–141	19.71	16	100.0	0.12<0.30	87.5	0.0067<0.01	P	37.5	0.32>0.30	100.0	0.0025<0.01	N	43.8	0.30=0.30	100.0	0.0031<0.01	S
50	Voutsas et al. in *Fluid Phase Equilib.* **2011**, 308, 135–141	32.86	14	85.7	0.33<0.49	78.6	0.0084<0.01	P	100.0	0.37<0.49	78.6	0.0079<0.01	P	78.6	0.42<0.49	71.4	0.0076<0.01	S
51	Othmer et al in Gmehling, J. et al. vol 1, part 1A, page 139	287.4	13	30.8	8.85>4.31	30.8	0.0190>0.01	N	7.7	15.49>4.31	61.5	0.0104>0.01	N	7.7	16.02>4.31	61.5	0.0105>0.01	N
52	Othmer et al in Gmehling, J. et al. vol 1, part 1, page 176	515.0	11	45.5	11.01>7.72	27.3	0.0260>0.01	N	72.7	13.09>7.72	18.2	0.0325>0.01	N	72.7	13.54>7.72	18.2	0.0316>0.01	N
53	Othmer et al in Gmehling, J. et al. vol 1, part 1, page 175	721.8	11	72.7	14.84>10.83	18.2	0.0361>0.01	N	0.0	59.00>10.83	72.7	0.0098<0.01	N	0.0	61.11>10.83	81.8	0.0082<0.01	N

Table S4. Results of consistency tests on isothermal VLE binary systems of ethanol (1) + water (2) using Antoine constants of this work (TW)

	VLE data			Fredenslund et al.[24] test					PTDGE				
	References	T/K	N data	%p>75	MAD p/kPa	%y_1>75	MAD y_1	TEST	%p>75	MAD p/kPa	%y_1>75	MAD y_1	TEST
1	Dulitskaya, K.A. in Gmehling, J. et al. vol 1, part 1, page 161	323.15	6	100.0	0.06<0.32	62.5	0.0193>0.01	N	0.0	2.38>0.32	83.3	0.0095<0.01	N
2	Jones et al. in Gmehling, J. et al. vol 1, part 1, page 163	323.65	9	90.9	0.17<0.33	72.7	0.0077<0.01	S	22.2	0.61>0.33	66.7	0.0095<0.01	N
3	Jones et al. in Gmehling, J. et al. vol 1, part 1, page 164	333.65	11	100.0	0.13<0.52	69.2	0.0086<0.01	S	36.4	0.66>0.52	81.8	0.0071<0.01	N
4	Kleinert, T. in Gmehling, J. et al. vol 1, part 1B, page 93	393.15	21	100.0	1.50<4.67	56.5	0.0120>0.01	N	28.6	6.76<4.67	76.2	0.0075<0.01	N
5	Kurihara et al. in Gmehling, J. et al. vol 1, part 1C, page 198−199	323.15	37	100.0	0.03<0.32	64.1	0.0080<0.01	S	18.9	0.39>0.32	86.5	0.0064<0.01	N
6	Kurihara et al. in Gmehling, J. et al. vol 1, part 1C, page 200	328.15	34	100.0	0.05<0.40	72.2	0.0076<0.01	S	38.2	0.43>0.40	85.3	0.0049<0.01	N
7	Kurihara et al. in Gmehling, J. et al. vol 1, part 1C, page 202−203	333.15	33	100.0	0.03<0.51	51.4	0.0108>0.01	N	33.3	0.59>0.51	63.6	0.0092<0.01	N
8	Mertl, I. in Gmehling, J. et al. vol 1, part 1, page 172	313.15	13	73.3	0.16<0.19	60.0	0.0143>0.01	N	7.7	0.51>0.19	84.6	0.0051<0.01	N
9	Mertl, I. in Gmehling, J. et al. vol 1, part 1, page 173	328.15	13	100.0	0.12<0.40	93.3	0.0040<0.01	P	84.6	0.34<0.40	100.0	0.0024<0.01	P
10	Mertl, I. in Gmehling, J. et al. vol 1, part 1, page 174	343.15	13	100.0	0.16<0.78	100.0	0.0021<0.01	P	100.0	0.14<0.78	100.0	0.0020<0.01	P
11	Nikolkaya, A.V. in Gmehling, J. et al. vol 1, part 1B, page 105	298.15	5	100.0	0.01<0.08	71.4	0.0147>0.01	N	40.0	0.47>0.08	20.0	0.0638>0.01	N
12	Nikolkaya, A.V. in Gmehling, J. et al. vol 1, part 1B, page 106	323.15	7	100.0	0.10<0.32	66.7	0.0159<0.01	N	0.0	1.82>0.32	71.4	0.0092<0.01	N
13	Nikolkaya, A.V. in Gmehling, J. et al. vol 1, part 1B, page 107	348.15	6	100.0	0.02<0.96	50.0	0.0132>0.01	N	16.7	3.24>0.96	100.0	0.0045<0.01	N
14	Phutela et al. in Gmehling, J. et al. vol 1, part 1B, page 108	298.14	12	100.0	0.00<0.08	42.9	0.0154>0.01	N	0.0	0.35>0.08	58.3	0.0108>0.01	N
15	Rao et al. in Gmehling, J. et al. vol 1, part 1A, page 147	338.15	17	89.5	0.36<0.63	47.4	0.0220>0.01	N	11.8	2.02>0.63	41.2	0.0188>0.01	N
16	Rao et al. in Gmehling, J. et al. vol 1, part 1A, page 148	343.15	17	94.7	0.41<0.78	47.4	0.0181>0.01	N	35.3	1.57>0.78	47.1	0.0146>0.01	N
17	Rao et al. in Gmehling, J. et al. vol 1, part 1A, page 149	348.15	9	81.8	0.73<0.96	45.5	0.0196>0.01	N	11.1	3.48>0.96	55.6	0.0136>0.01	N
18	Rao et al. in Gmehling, J. et al. vol 1, part 1A, page 150	353.15	12	92.9	0.60<1.17	64.3	0.0158>0.01	N	41.7	3.21>1.17	25.0	0.0241>0.01	N
19	Udovenko&Fatkulina in Gmehling, J. et al. vol 1, part 1, page 190	313.15	10	83.3	0.11<0.19	75.0	0.0131>0.01	N	40.0	0.31>0.19	60.0	0.0162>0.01	N
20	Udovenko&Fatkulina in Gmehling, J. et al. vol 1, part 1, page 191	323.15	10	91.7	0.11<0.32	75.0	0.0199>0.01	N	50.0	0.51>0.32	70.0	0.0251>0.01	N
21	Udovenko&Fatkulina in Gmehling, J. et al. vol 1, part 1, page 192	333.15	10	100.0	0.13<0.51	66.7	0.0215>0.01	N	40.0	0.94>0.51	50.0	0.0245>0.01	N
22	Vrevsky, M.S. in Gmehling, J. et al. vol 1, part 1, page 194	312.91	16	100.0	0.04<0.19	27.8	0.0395>0.01	N	0.0	1.58>0.19	25.0	0.0184>0.01	N
23	Vrevsky, M.S. in Gmehling, J. et al. vol 1, part 1, page 195	327.96	9	100.0	0.05<0.40	45.5	0.0255>0.01	N	0.0	2.66>0.40	77.8	0.0109>0.01	N
24	Vrevsky, M.S. in Gmehling, J. et al. vol 1, part 1, page 196	347.94	12	100.0	0.30<0.95	28.6	0.0204>0.01	N	0.0	4.15>0.95	66.7	0.0109>0.01	N
25	Yamamoto et al. in Gmehling, J. et al. vol 1, part 1C, page 150	298.15	11	100.0	0.03<0.08	23.1	0.0238>0.01	N	9.1	0.29>0.08	18.2	0.0227>0.01	N
26	Arango&Villa in *Fluid Phase Equilib.* **2013**, 339, 31−39	353.15	10	91.7	0.58<1.17	41.7	0.0233>0.01	N	20.0	3.71>1.17	20.0	0.0243>0.01	N
27	Arango&Villa in *Fluid Phase Equilib.* **2013**, 339, 31−39	363.15	10	100.0	0.39<1.71	50.0	0.0188>0.01	N	30.0	3.17>1.71	20.0	0.0298>0.01	N

Table S5. Results of consistency tests on isothermal VLE binary systems of ethanol (1) + water (2) using Antoine constants of D&D[22]

	VLE data			Fredenslund et al.[24] test					PTDGE				
	References	T/K	N data	%p>75	MAD p/kPa	%y_1>75	MAD y_1	TEST	%p>75	MAD p/kPa	%y_1>75	MAD y_1	TEST
1	Dulitskaya, K.A. in Gmehling, J. et al. vol 1, part 1, page 161	323.15	6	100.0	0.02<0.32	62.5	0.0114>0.01	N	33.3	0.88>0.32	83.3	0.0087<0.01	N
2	Jones et al. in Gmehling, J. et al. vol 1, part 1, page 163	323.65	9	90.9	0.19<0.33	72.7	0.0106>0.01	N	33.3	0.63>0.33	55.6	0.0097<0.01	N
3	Jones et al. in Gmehling, J. et al. vol 1, part 1, page 164	333.65	11	100.0	0.15<0.52	38.5	0.0165>0.01	N	9.1	1.93>0.52	81.8	0.0085<0.01	N
4	Kleinert, T. in Gmehling, J. et al. vol 1, part 1B, page 93	393.15	21	95.7	1.39<4.67	56.5	0.0124>0.01	N	28.6	6.40>4.67	81.0	0.0071<0.01	N
5	Kurihara et al. in Gmehling, J. et al. vol 1, part 1C, page 198−199	323.15	37	94.9	0.11<0.32	76.9	0.0058<0.01	P	0.0	0.65>0.32	86.5	0.0053<0.01	N
6	Kurihara et al. in Gmehling, J. et al. vol 1, part 1C, page 200	328.15	34	97.2	0.09<0.40	88.9	0.0057<0.01	P	0.0	0.70>0.40	91.2	0.0040<0.01	N
7	Kurihara et al. in Gmehling, J. et al. vol 1, part 1C, page 202−203	333.15	33	100.0	0.08<0.51	91.4	0.0071<0.01	P	30.3	0.61>0.51	72.7	0.0079<0.01	N
8	Mertl, I. in Gmehling, J. et al. vol 1, part 1, page 172	313.15	13	53.3	0.24>0.19	60.0	0.0089<0.01	N	7.7	0.61>0.19	84.6	0.0041<0.01	N
9	Mertl, I. in Gmehling, J. et al. vol 1, part 1, page 173	328.15	13	100.0	0.12<0.40	80.0	0.0086<0.01	P	7.7	0.90>0.40	100.0	0.0020<0.01	N
10	Mertl, I. in Gmehling, J. et al. vol 1, part 1, page 174	343.15	13	100.0	0.15<0.78	73.3	0.0082<0.01	S	0.0	1.39>0.78	100.0	0.0025<0.01	N
11	Nikolkaya, A.V. in Gmehling, J. et al. vol 1, part 1B, page 105	298.15	5	85.7	0.12>0.08	57.1	0.0096<0.01	N	0.0	0.97>0.08	20.0	0.0644>0.01	N
12	Nikolkaya, A.V. in Gmehling, J. et al. vol 1, part 1B, page 106	323.15	7	100.0	0.08<0.32	77.8	0.0092<0.01	P	57.1	0.54>0.32	85.7	0.0082<0.01	N
13	Nikolkaya, A.V. in Gmehling, J. et al. vol 1, part 1B, page 107	348.15	6	100.0	0.08<0.96	87.5	0.0074<0.01	P	33.3	1.65>0.96	100.0	0.0035<0.01	N
14	Phutela et al. in Gmehling, J. et al. vol 1, part 1B, page 108	298.14	12	100.0	0.01<0.08	92.9	0.0029<0.01	P	0.0	0.28>0.08	66.7	0.0099<0.01	N
15	Rao et al. in Gmehling, J. et al. vol 1, part 1A, page 147	338.15	17	89.5	0.38<0.63	42.1	0.0175>0.01	N	52.9	0.77>0.63	47.1	0.0186>0.01	N
16	Rao et al. in Gmehling, J. et al. vol 1, part 1A, page 148	343.15	17	100.0	0.36<0.78	42.1	0.0166>0.01	N	35.3	1.18>0.78	47.1	0.0150>0.01	N
17	Rao et al. in Gmehling, J. et al. vol 1, part 1A, page 149	348.15	9	81.8	0.73<0.96	45.5	0.0152>0.01	N	11.1	2.20>0.96	55.6	0.0123>0.01	N
18	Rao et al. in Gmehling, J. et al. vol 1, part 1A, page 150	353.15	12	92.9	0.56<1.17	57.1	0.0162>0.01	N	16.7	2.65>1.17	25.0	0.0242>0.01	N
19	Udovenko&Fatkulina in Gmehling, J. et al. vol 1, part 1, page 190	313.15	10	100.0	0.05<0.19	33.3	0.0243>0.01	N	10.0	1.00>0.19	50.0	0.0170>0.01	N
20	Udovenko&Fatkulina in Gmehling, J. et al. vol 1, part 1, page 191	323.15	10	100.0	0.11<0.32	25.0	0.0346>0.01	N	10.0	1.52>0.32	60.0	0.0264>0.01	N
21	Udovenko&Fatkulina in Gmehling, J. et al. vol 1, part 1, page 192	333.15	10	100.0	0.13<0.51	25.0	0.0326>0.01	N	10.0	2.13>0.51	30.0	0.0259>0.01	N
22	Vrevsky, M.S. in Gmehling, J. et al. vol 1, part 1, page 194	312.91	16	94.4	0.08<0.19	38.9	0.0258>0.01	N	18.8	0.77>0.19	25.0	0.0173>0.01	N
23	Vrevsky, M.S. in Gmehling, J. et al. vol 1, part 1, page 195	327.96	9	100.0	0.07<0.40	63.6	0.0134>0.01	N	11.1	1.26>0.40	88.9	0.0096<0.01	N
24	Vrevsky, M.S. in Gmehling, J. et al. vol 1, part 1, page 196	347.94	12	100.0	0.24<0.95	57.1	0.0138>0.01	N	0.0	2.57>0.95	75.0	0.0093<0.01	N
25	Yamamoto et al. in Gmehling, J. et al. vol 1, part 1C, page 150	298.15	11	69.2	0.07<0.08	76.9	0.0089<0.01	S	9.1	0.34>0.08	18.2	0.0215>0.01	N
26	Arango&Villa in *Fluid Phase Equilib.* **2013**, 339, 31−39	353.15	10	91.7	0.61<1.17	41.7	0.0193>0.01	N	30.0	2.68>1.17	20.0	0.0226>0.01	N
27	Arango&Villa in *Fluid Phase Equilib.* **2013**, 339, 31−39	363.15	10	100.0	0.41<1.71	58.3	0.0158>0.01	N	20.0	4.10>1.71	20.0	0.0281>0.01	N

Table S6. Results of consistency tests on isothermal VLE binary systems of ethanol (1) + water (2) using Antoine constants of VDI[23]

	VLE data			Fredenslund et al.[24] test					PTDGE				
	References	T/K	N data	%p>75	MAD p/kPa	%y_1>75	MAD y_1	TEST	%p>75	MAD p/kPa	%y_1>75	MAD y_1	TEST
1	Dulitskaya, K.A. in Gmehling, J. et al. vol 1, part 1, page 161	323.15	6	100.0	0.02<0.32	75.0	0.0120>0.01	N	0.0	1.37>0.32	100.0	0.0040<0.01	N
2	Jones et al. in Gmehling, J. et al. vol 1, part 1, page 163	323.65	9	81.8	0.17<0.33	54.5	0.0102>0.01	N	55.6	0.41>0.33	44.4	0.0132>0.01	N
3	Jones et al. in Gmehling, J. et al. vol 1, part 1, page 164	333.65	11	100.0	0.13<0.52	46.2	0.0142>0.01	N	18.2	0.94>0.52	45.5	0.0134>0.01	N
4	Kleinert, T. in Gmehling, J. et al. vol 1, part 1B, page 93	393.15	21	95.7	1.47<4.67	56.5	0.0121>0.01	N	28.6	6.89>4.67	76.2	0.0074<0.01	N
5	Kurihara et al. in Gmehling, J. et al. vol 1, part 1C, page 198−199	323.15	37	100.0	0.07<0.32	100.0	0.0035<0.01	P	97.3	0.28<0.32	100.0	0.0012<0.01	P
6	Kurihara et al. in Gmehling, J. et al. vol 1, part 1C, page 200	328.15	34	100.0	0.07<0.40	100.0	0.0034<0.01	P	97.1	0.38<0.40	100.0	0.0015<0.01	P
7	Kurihara et al. in Gmehling, J. et al. vol 1, part 1C, page 202−203	333.15	33	100.0	0.10<0.51	82.9	0.0067<0.01	P	97.0	0.43>0.51	87.9	0.0040<0.01	P
8	Mertl, I. in Gmehling, J. et al. vol 1, part 1, page 172	313.15	13	80.0	0.15<0.19	46.7	0.0133>0.01	N	53.8	0.29>0.19	92.3	0.0053<0.01	N
9	Mertl, I. in Gmehling, J. et al. vol 1, part 1, page 173	328.15	13	100.0	0.13<0.40	93.3	0.0040<0.01	P	84.6	0.22<0.40	100.0	0.0057<0.01	P
10	Mertl, I. in Gmehling, J. et al. vol 1, part 1, page 174	343.15	13	100.0	0.04<0.78	80.0	0.0064<0.01	P	92.3	0.58<0.78	100.0	0.0051<0.01	P
11	Nikolkaya, A.V. in Gmehling, J. et al. vol 1, part 1B, page 105	298.15	5	85.7	0.03<0.08	57.1	0.0125>0.01	N	20.0	0.57>0.08	20.0	0.0717>0.01	N
12	Nikolkaya, A.V. in Gmehling, J. et al. vol 1, part 1B, page 106	323.15	7	100.0	0.10<0.32	77.8	0.0104>0.01	N	0.0	1.10>0.32	100.0	0.0045<0.01	N
13	Nikolkaya, A.V. in Gmehling, J. et al. vol 1, part 1B, page 107	348.15	6	100.0	0.13<0.96	87.5	0.0081<0.01	P	33.3	1.82>0.96	100.0	0.0034<0.01	N
14	Phutela et al. in Gmehling, J. et al. vol 1, part 1B, page 108	298.14	12	100.0	0.02<0.08	100.0	0.0054<0.01	P	16.7	0.16>0.08	91.7	0.0042<0.01	N
15	Rao et al. in Gmehling, J. et al. vol 1, part 1A, page 147	338.15	17	84.2	0.38<0.63	42.1	0.0173>0.01	N	29.4	1.04>0.63	47.1	0.0181>0.01	N
16	Rao et al. in Gmehling, J. et al. vol 1, part 1A, page 148	343.15	17	100.0	0.36<0.78	42.1	0.0167>0.01	N	42.1	1.15>0.78	41.2	0.0158>0.01	N
17	Rao et al. in Gmehling, J. et al. vol 1, part 1A, page 149	348.15	9	81.8	0.72<0.96	45.5	0.0151>0.01	N	11.1	2.72>0.96	55.6	0.0105>0.01	N
18	Rao et al. in Gmehling, J. et al. vol 1, part 1A, page 150	353.15	12	92.9	0.57<1.17	64.3	0.0161>0.01	N	25.0	2.68>1.17	25.0	0.0242>0.01	N
19	Udovenko&Fatkulina in Gmehling, J. et al. vol 1, part 1, page 190	313.15	10	100.0	0.03<0.19	50.0	0.0211>0.01	N	30.0	0.49>0.19	20.0	0.0230>0.01	N
20	Udovenko&Fatkulina in Gmehling, J. et al. vol 1, part 1, page 191	323.15	10	100.0	0.09<0.32	58.3	0.0267>0.01	N	40.0	0.57>0.32	40.0	0.0325>0.01	N
21	Udovenko&Fatkulina in Gmehling, J. et al. vol 1, part 1, page 192	333.15	10	100.0	0.13<0.51	58.3	0.0268>0.01	N	50.0	1.04>0.51	30.0	0.0302>0.01	N
22	Vrevsky, M.S. in Gmehling, J. et al. vol 1, part 1, page 194	312.91	16	100.0	0.04<0.19	33.3	0.0259>0.01	N	0.0	1.14>0.19	68.8	0.0098<0.01	N
23	Vrevsky, M.S. in Gmehling, J. et al. vol 1, part 1, page 195	327.96	9	100.0	0.09<0.40	63.6	0.0151>0.01	N	0.0	1.82>0.40	100.0	0.0051<0.01	N
24	Vrevsky, M.S. in Gmehling, J. et al. vol 1, part 1, page 196	347.94	12	100.0	0.32<0.95	50.0	0.0147>0.01	N	0.0	3.16>0.95	91.7	0.0073<0.01	N
25	Yamamoto et al. in Gmehling, J. et al. vol 1, part 1C, page 150	298.15	11	100.0	0.03<0.08	76.8	0.0087<0.01	P	54.5	0.12>0.08	63.6	0.0082<0.01	N
26	Arango&Villa in *Fluid Phase Equilib.* **2013**, 339, 31−39	353.15	10	100.0	0.57<1.17	41.7	0.0192>0.01	N	30.0	2.95>1.17	40.0	0.0212>0.01	N
27	Arango&Villa in *Fluid Phase Equilib.* **2013**, 339, 31−39	363.15	10	100.0	0.40<1.71	58.3	0.0158>0.01	N	20.0	3.91>1.71	10.0	0.0277>0.01	N
